# NOX2 inhibition attenuates oxidative stress and eNOS uncoupling in pulmonary arteries of rats following simulated air diving

**DOI:** 10.1371/journal.pone.0351145

**Published:** 2026-07-02

**Authors:** Qingbo Gong, Lin Xiao, Song Wang

**Affiliations:** 1 School of Sports Medicine, Wuhan Sports University, Wuhan, China; 2 Department of Physical Education, Chongqing University of Arts and Sciences, Chongqing, China; 3 Department of Physical Education and Health, Zhaoqing University, Zhaoqing, China; 4 Sports Science and Technology College of Wuhan Sports University, Wuhan, China; Longgang Otorhinolaryngology Hospital & Shenzhen Key Laboratory of Otorhinolaryngology, Shenzhen Institute of Otorhinolaryngology, CHINA

## Abstract

Simulated diving and decompression can impair endothelial function, but the upstream oxidant sources and their relationship with endothelial nitric oxide synthase (eNOS) coupling in the pulmonary circulation remain unclear. We investigated whether NADPH oxidase 2 (NOX2) is associated with oxidative stress, tetrahydrobiopterin (BH4) depletion, altered eNOS coupling, and pulmonary endothelial dysfunction after simulated air diving. Eighteen male Sprague–Dawley rats were assigned to three groups: control, decompression stress, and decompression stress with the NOX2 inhibitor GSK2795039 (100 mg/kg, intraperitoneal) administered before pressurization. Decompression stress was induced by hyperbaric exposure to 600 kPa for 1 h followed by decompression to ambient pressure; pulmonary arteries were collected 1 h after decompression. We evaluated NOX2 expression, oxidative stress indices, BH4 content, eNOS phosphorylation and dimer/monomer ratio, nitric oxide metabolites (nitrate plus nitrite), markers associated with endothelial activation, and vasoreactivity. Compared with controls, decompression stress increased NOX2 expression, reactive oxygen species and lipid peroxidation, decreased superoxide dismutase activity, reduced BH4 and nitric oxide metabolites. It also caused a shift in eNOS towards a lower dimer/monomer ratio, increased endothelin-1 and adhesion molecules, and impaired endothelium-dependent relaxation, though endothelium-independent relaxation remained intact. GSK2795039 pretreatment attenuated oxidative stress, improved BH4 availability, restored nitric oxide metabolites, and decreased markers of endothelial activation, partially improving endothelium-dependent relaxation. These findings suggest that NOX2-associated oxidative stress contributes to reduced BH4 availability and eNOS coupling imbalance, leading to pulmonary endothelial dysfunction after decompression.

## Introduction

Scuba diving entails hyperbaric exposure followed by decompression, which can lead to decompression sickness (DCS). Although the incidence rate is relatively low (~0.01–0.095% of dives), endothelial injury constitutes a central feature of DCS pathophysiology [1]. Post-dive studies in both divers and animal models have reported impaired endothelial function, often accompanied by reduced nitric oxide (NO) bioavailability and elevated oxidative stress [2,3]. While intravascular bubble formation during decompression is a hallmark of DCS, growing evidence suggests that oxidative stress and reactive oxygen species (ROS) overproduction may contribute to endothelial damage, even when vascular gas emboli (VGE) are minimal or undetectable [4,5]. This implies that bubble load alone may not fully account for vascular injury.

During decompression, intravascular bubbles can stimulate the vascular wall, generate mechanical stimuli and promote inflammatory activation [6]. These stimuli have been linked to the activation of NADPH oxidases (NOX), a major enzymatic source of ROS in vascular cells [7]. Among NOX isoforms, NOX2 has been implicated in pathological ROS production in endothelial dysfunction and vascular disease [8]. Excess ROS can oxidize tetrahydrobiopterin (BH4), a critical cofactor for eNOS, leading to uncoupling [9]. Once uncoupled, eNOS generates superoxide rather than NO, amplifying oxidative stress and reducing NO bioavailability [10]. This redox-dependent feed-forward mechanism is well documented in chronic cardiovascular diseases such as hypertension and atherosclerosis [11,12].

Endothelial dysfunction and oxidative stress have been reported in divers and experimental models of decompression [3,13]. However, the role of NOX2-related oxidative signaling in pulmonary arteries, particularly its connection to BH4 availability and eNOS coupling after decompression is unclear. Current research often focuses on systemic oxidative markers or general antioxidants [5,14,15], neglecting NOX2 pathways and their link to eNOS coupling. Thus, it is crucial to investigate if uncoupling occurs during acute decompression stress and how it relates to NOX2-associated oxidative stress.

In this study, we tested the hypothesis that NOX2 activation during simulated air diving contributes to oxidative stress and affects BH4 availability and eNOS coupling in pulmonary arteries. We investigated whether the NOX2 inhibitor GSK2795039 could mitigate these effects and enhance endothelial function. Using a rat model, we combined biochemical assays and wire-myography to assess oxidative stress, BH4 levels, eNOS coupling, and vascular reactivity. Specifically, we predicted that NOX2 inhibition would attenuate oxidative stress, preserve BH4 availability, and restore eNOS coupling, thereby improving endothelium-dependent relaxation.

## Materials and methods

### Ethics statement

Eighteen male Sprague–Dawley rats (280–320 g) were obtained from Hubei Laboratory Animal Center (License No. SCXK (E) 20250018). Rats were housed under controlled temperature (22 ± 2°C), humidity (50 ± 10%), and a 12-hour light/dark cycle. They had free access to standard chow and water and were acclimatized for at least one week before experiments. All procedures followed NIH guidelines and were approved by the Ethics Committee of Wuhan Sports University (No. 0087-202110-0101).

Rats were deeply anesthetized with sodium pentobarbital (50 mg/kg, i.p.) and euthanized by exsanguination followed by thoracotomy to ensure humane treatment. Death was confirmed by the absence of heartbeat and breathing. To reduce distress, animals were acclimated for a week and monitored daily. During and after simulated diving, animals were closely observed for signs of distress or neurological abnormalities. If persistent pain or severe distress had been observed, buprenorphine or immediate euthanasia would have been administered. However, no unexpected deaths occurred and no humane endpoints were met; therefore, buprenorphine was not used.

### Experiment grouping

Eighteen male rats were randomly allocated into three groups (n = 6 per group): Control group (Group C): The animals were placed in the hyperbaric chamber at ambient pressure (100 kPa; 1 atmosphere absolute [ATA]) for a duration equivalent to the simulated diving protocol but without compression. This procedure controlled for any stress associated with chamber confinement. Rats received a single intraperitoneal (i.p.) injection of vehicle (volume-matched) 30 minutes before exposure. Decompression-stress group (Group D): Rats underwent a simulated air-diving and decompression protocol in a hyperbaric chamber, as described in the “Simulated diving procedure” section. Rats received a single i.p. injection of vehicle (volume-matched) 30 minutes before pressurization. NOX2 inhibitor–treated decompression-stress group (Group D + GSK2795039): The animals received a single i.p. dose of GSK2795039 (100 mg/kg; MedChem Express, NJ, USA; Cat. No. HY-18950) 30 minutes before pressurization, followed by the same protocol as the decompression-stress group. All injections were administered once before chamber exposure.

### Simulated diving procedure

Animals were pressurized in a chamber to 600 kPa at a rate of 100 kPa/min, maintained for 1 hour, then decompressed at a rate of 200 kPa/min. The chamber was ventilated for 5 minutes every 15 minutes to prevent CO₂ buildup. After decompression, animals were continuously observed for behavioral and neurological abnormalities (e.g., labored breathing, tremor, paralysis, or prolonged recumbency) until tissue collection at 60 minutes post-decompression.

### Tissue collection

Pulmonary arteries were collected 1 hour after decompression (Group D and Group D + GSK2795039) or 1 hour after chamber exposure at ambient pressure (Group C). Sample collection and processing followed established rodent decompression protocols [16,17]. Vessels were cleaned of surrounding connective tissue in ice-cold physiological buffer. For vascular reactivity experiments, arteries were cut into rings and mounted immediately for isometric tension recording. Consistent with prior decompression studies, endothelial function was evaluated in intact pulmonary artery rings by acetylcholine-induced endothelium-dependent relaxation, while sodium nitroprusside was used to assess endothelium-independent relaxation [16,17]. For biochemical assays, ELISA, and Western blotting, arterial tissue was snap-frozen in liquid nitrogen and stored at −80°C. Because decompression-related studies typically quantify oxidative stress and endothelial activation markers in serum or whole-vessel homogenates rather than in isolated endothelial cells [16,18–20], biochemical and ELISA assays in the present study were performed on homogenates of intact pulmonary artery segments. These outcomes are therefore interpreted as vessel-level indices consistent with endothelial dysfunction or activation, recognizing that they are not endothelial cell–specific and may include contributions from non-endothelial vascular components [16,19].

### Sample size calculation

Sample size was predetermined for two key outcomes: (i) endothelium-dependent relaxation in pulmonary artery rings (primary functional outcome) and (ii) the eNOS dimer/monomer ratio (key mechanistic outcome). Sample size calculations were performed using the standard equation for two independent means, as described by Clifton [21].


$$\begin{array}{c} n=\frac{(Z_{1-\alpha /2}+Z_{1-\beta }{)}^{2}\cdot 2\sigma^{2}}{\delta^{2}} \end{array}$$

where *n* is the required sample size per group, α is the two-sided significance level, β corresponds to statistical power, Z_1-α/2_ and Z_1-β_ are the standard normal quantiles, σ is the pooled standard deviation, and δ is the expected difference in group means. Using α = 0.05 and power = 0.80 (β = 0.20), pilot data for maximal vascular ring relaxation (Emax) yielded 𝜎 = 5.83 and δ = 19.30, giving an estimated n = 1.43 animals per group. Given biological/technical variability and potential tissue loss during ring preparation, we conservatively used n = 3 animals per group for vascular ring experiments. For the eNOS dimer/monomer ratio, pilot data yielded σ = 2.21 and δ = 4.03, giving an estimated n = 4.72; therefore, we used n = 6 animals per group for this endpoint. Accordingly, vascular endothelial function experiments were performed with n = 3 animals per group, whereas all other measurements were performed with n = 6 animals per group, unless otherwise specified.

### Western blot

Pulmonary artery protein levels of NOX2, total eNOS, phospho-eNOS (Ser1177), and the eNOS dimer/monomer ratio were assessed by Western blotting. The frozen arteries were homogenized and lysed in RIPA lysis buffer (Wuhan Servicebio Technology Co., Ltd., Wuhan, China; Cat. No. G2002) supplemented with protease and phosphatase inhibitor cocktails (Servicebio; Cat. No. G2006 and G2007, respectively). Lysates were centrifuged at 12,000 × g for 10 minutes at 4°C, and supernatants were assayed for protein concentration using a BCA kit (Nanjing Jiancheng Bioengineering Institute, Nanjing, China; Cat. No. A045-4). Equal amounts of protein (30 μg per lane) were separated by SDS‒PAGE on 8% Tris-glycine gels (Servicebio; Cat. No. G2003) and transferred onto a PVDF membrane (0.45 μm; Servicebio; Cat. No. G6015-0.45). Membranes were blocked in TBST [TBS (Cat. No. G0001-2L) with Tween-20 (Cat. No. WGT8220); Servicebio] containing 5% skim milk (Cat. No. G5002; Servicebio) for 30 minutes, or with 5% BSA for phospho-specific antibodies, and then incubated with primary antibodies overnight at 4°C. Primary antibodies (all from Wuhan Fine Biotech Co., Ltd., Wuhan, China; hereafter Fine Biotech) included: NOX2 (Cat. No. FNab05804; 1:1000), eNOS (Cat. No. FNab10162; 1:1000), phospho-eNOS (Ser1177) (Cat. No. FNab10931; 1:500), and GAPDH (Cat. No. FNab03343; 1:5000). After washing, membranes were incubated with HRP-conjugated secondary antibodies (goat anti-rabbit IgG, Cat. No. GB23303, 1:5000; goat anti-mouse IgG, Cat. No. GB23301, 1:5000; Servicebio) for 1 hour at room temperature. Protein bands were visualized using an ECL reagent (Cat. No. G2014; Servicebio) and imaged using a Kodak In-Vivo Imaging System. Band intensities were quantified using ImageJ and normalized to GAPDH.

For eNOS dimer/monomer analysis, total eNOS was detected using the same eNOS antibody (Cat. No. FNab10162; Fine Biotech) under non‑reducing conditions (no reducing agent, no boiling) and low-temperature electrophoresis to preserve the dimer form.

### Biochemical assays

#### Reactive oxygen species (ROS) assay.

Pulmonary arteries were minced and gently dissociated through a 300-mesh nylon filter in ice-cold PBS to yield a cell suspension. Cells were pelleted at 500 × g for 10 min, washed once with PBS, and incubated with DCFH-DA fluorescent probe (Cat. No. E004-1–1; Nanjing Jiancheng Bioengineering Institute, Nanjing, China) at 37 °C for 1 hour. After centrifugation at 1,000 × g for 5 minutes, the pellet was washed twice with PBS, recovered by centrifugation, and resuspended in PBS. Fluorescence intensity was recorded at excitation/emission wavelengths of 500/525 nm and normalized to total protein concentration determined by BCA assay. Results were expressed as fluorescence intensity per mg of protein. Because DCFH-DA fluorescence reflects general oxidant load and is not specific to any single enzymatic source of ROS, this assay cannot differentiate among individual ROS-generating enzymes.

#### Superoxide dismutase (SOD) assay.

Pulmonary arteries were dissected into small fragments, and lysis buffer from the SOD activity kit was added at a ratio of 100 μL per 10 mg of tissue. The tissue was homogenized on ice and centrifuged at 10,000 × g for 10 minutes at 4°C. The supernatant was collected for SOD activity measurement. SOD activity was evaluated using a commercial SOD activity assay kit (hydroxylamine method; Nanjing Jiancheng Bioengineering Institute, Nanjing, China; Cat. No. A001-1) according to the manufacturer’s instructions. This method is based on the ability of SOD to inhibit the reduction of a superoxide-sensitive substrate. Briefly, the tissue supernatant and assay reagents were added to a 96-well plate in the order and volumes specified by the kit, incubated at 37°C for the indicated time. Absorbance was measured at 550 nm using a microplate reader. Subsequently, SOD activity was calculated using the formula provided by the manufacturer and expressed as units per mg of protein (U/mg protein).

#### Malondialdehyde (MDA) assay.

Pulmonary arteries were dissected into small fragments, and the supplied MDA lysis buffer was added at a ratio of 100 μL per 10 mg of tissue. Tissue was homogenized on ice until complete lysis and centrifuged at 10,000 × g for 10 minutes at 4°C. The supernatant was collected for analysis. MDA levels were measured using a commercial assay kit (TBA method; Nanjing Jiancheng Bioengineering Institute, Nanjing, China; Cat. No. A003-1–2) according to the manufacturer’s instructions. Briefly, appropriate volumes of tissue supernatant and working reagent were mixed in microcentrifuge tubes, heated at the recommended temperature for the specified time, and then cooled on ice. The reaction mixtures were transferred to a 96-well plate, and absorbance was measured at 532 nm using a microplate reader. MDA content was calculated using the formula provided by the manufacturer and expressed as nmol per mg of protein.

#### NOx (total nitrate/nitrite) assay.

Pulmonary arteries were dissected into small fragments, and the lysis buffer was added at a ratio of 100 μL per 10 mg of tissue. Tissue was homogenized on ice until complete lysis. The lysate was centrifuged at 10,000 × g for 5 minutes, and the supernatant was collected. NOx (total nitrate/nitrite) levels were quantified using a commercial NO detection kit (Nanjing Jiancheng Bioengineering Institute, Nanjing, China; Cat. No. A012-1) according to the manufacturer's instructions. Absorbance was measured at 540 nm on a microplate reader. NOx content was calculated using the formula provided by the manufacturer and expressed as μmol per mg of protein.

### Assessment of vascular endothelial function

Pulmonary artery rings (approximately 2 mm in length) were carefully isolated and mounted in a wire myograph system (DMT110P, Aarhus N, Denmark) containing Krebs-Henseleit solution bubbled with 95% O_2_ and 5% CO_2_ at 37°C. Rings were equilibrated for 60 min at a resting tension of 10 mN, and contractile responsiveness was confirmed by exposure to 60 mM KCl. Rings were pre-contracted with phenylephrine (PE, 10 μM; MedChem Express, NJ, USA; Cat. No. HY-B0769) until a stable plateau was reached. Subsequently, endothelium-dependent relaxation was induced by cumulative addition of acetylcholine (ACh, 10 ⁻ ⁹ to 10 ⁻ ⁴ M; MedChem Express, NJ, USA; Cat. No. HY-B0282). Endothelium-independent relaxation was evaluated using sodium nitroprusside (SNP, 10 ⁻ ⁹ to 10 ⁻ ⁴ M; MedChem Express, NJ, USA; Cat. No. HY-B0564). Relaxation was expressed as a percentage of PE-induced pre-contraction. The data were analyzed using LabChart software.

#### ELISA assay.

Pulmonary artery tissue was homogenized in lysis buffer (100 μL per 10 mg of tissue) on ice and centrifuged at 10,000 × g for 10 minutes at 4°C. The supernatant was collected for subsequent analysis. Total protein concentration was determined using a BCA protein assay.

Endothelin-1 (ET-1), vascular cell adhesion molecule-1 (VCAM-1), intercellular adhesion molecule-1 (ICAM-1), and tetrahydrobiopterin (BH4) were measured using commercial rat ELISA kits according to the manufacturers’ instructions. The kits used were as follows: ET-1 (Wuhan Fine Biotech, Wuhan, China; Cat. No. ER0019), VCAM-1 (Fine Biotech; Cat. No. ER1358), ICAM-1 (Fine Biotech; Cat. No. ER0028), and BH4 (Shanghai Coibo Biotechnology, Shanghai, China; Cat. No. CB10776-Ra). Briefly, 100 μL of standards or samples was added to the pre-coated 96-well plate and incubated at 37°C for 90 minutes. After washing, 100 μL of biotinylated detection antibody was added and incubated at 37°C for 60 minutes. The plates were washed and incubated with 100 μL of HRP–streptavidin solution at 37°C for 30 minutes. After washing, 90 μL of TMB substrate was added and incubated at 37°C for 15 minutes in the dark. The reaction was stopped by adding 50 μL of stop solution, and absorbance was measured immediately at 450 nm. For ELISA quantification, a standard curve was generated by plotting the optical density (OD) at 450 nm against the corresponding concentrations of the standards. Sample concentrations were calculated by interpolation from the standard curve.

### Data analysis

The normality of the data was tested using the Shapiro‒Wilk test, and the homogeneity of variances was assessed using Levene's test. ANOVA was used to compare outcome data among groups when data were normally distributed, followed by Tukey's post hoc test for pairwise comparisons when a significant F-ratio was observed. For non-normally distributed data, group comparisons were conducted using the Kruskal–Wallis test. When significant, Bonferroni-corrected pairwise comparisons were performed. For vascular concentration–response curves, data were analyzed using two-way repeated-measures ANOVA (group × concentration), followed by Bonferroni-adjusted post hoc tests when appropriate. The significance level was set at *p* < 0.05. Statistical analysis was performed using IBM SPSS Statistics 26.0 and GraphPad Prism 9.0.

## Results

### Decompression-induced NOX2 upregulation in pulmonary arteries is attenuated by GSK2795039

**Fig 1:**
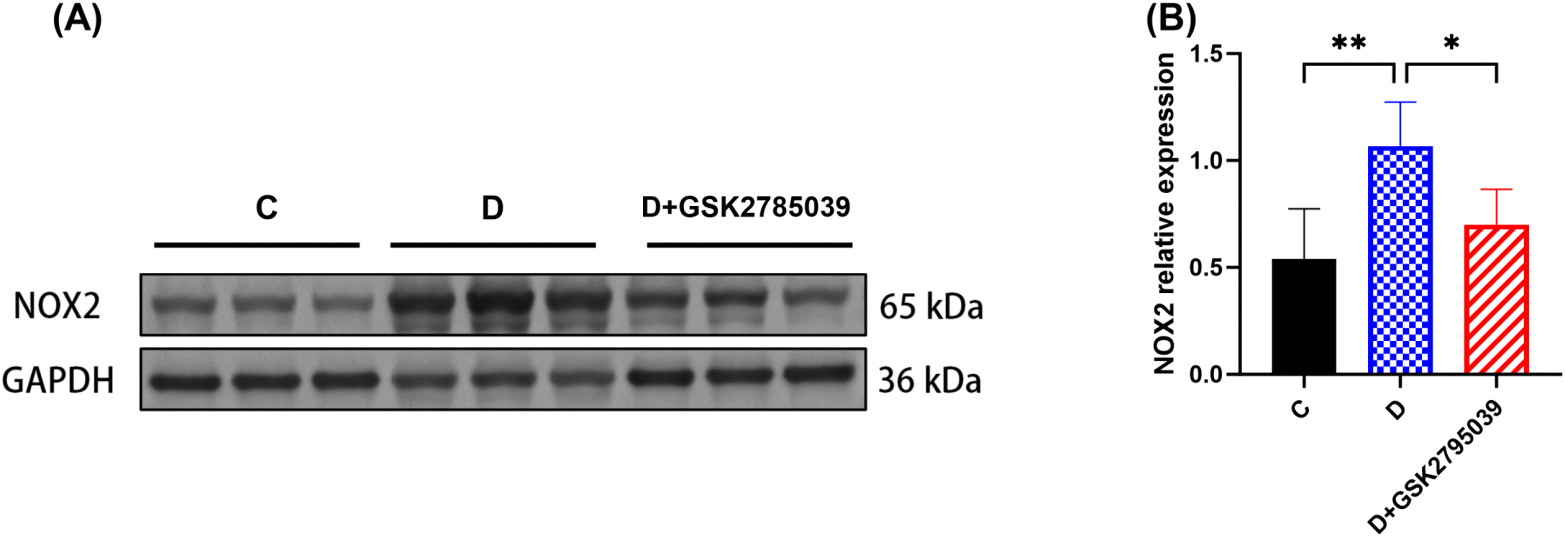
Decompression-induced NOX2 upregulation in pulmonary arteries is attenuated by GSK2795039. (A) Representative Western blots of NOX2 and GAPDH in pulmonary artery tissue homogenates from the Group C, Group D, and Group D + GSK2795039. (B) Densitometric quantification of NOX2 protein expression normalized to GAPDH. Data are presented as mean ± SD (n = 6 per group). Statistical analysis was performed using one-way ANOVA followed by Tukey's post hoc test. * *p* < 0.05, ** *p* < 0.01, *** *p* < 0.001, **** *p* < 0.0001.

To evaluate whether NOX2 is involved in decompression-related vascular changes, we measured NOX2 protein expression in pulmonary arteries (whole-vessel homogenates). NOX2 expression was significantly increased in Group D compared with Group C (Fig 1A, B; *p* = 0.0012). Pretreatment with the NOX2 inhibitor GSK2795039 was associated with a significant reduction in NOX2 expression compared with Group D (Fig 1A, B; *p* = 0.0018), restoring it to a level not different from Group C (*p* = 0.3784).

### GSK2795039 pretreatment attenuates decompression-associated changes in oxidative stress indices in pulmonary arteries

**Fig 2:**
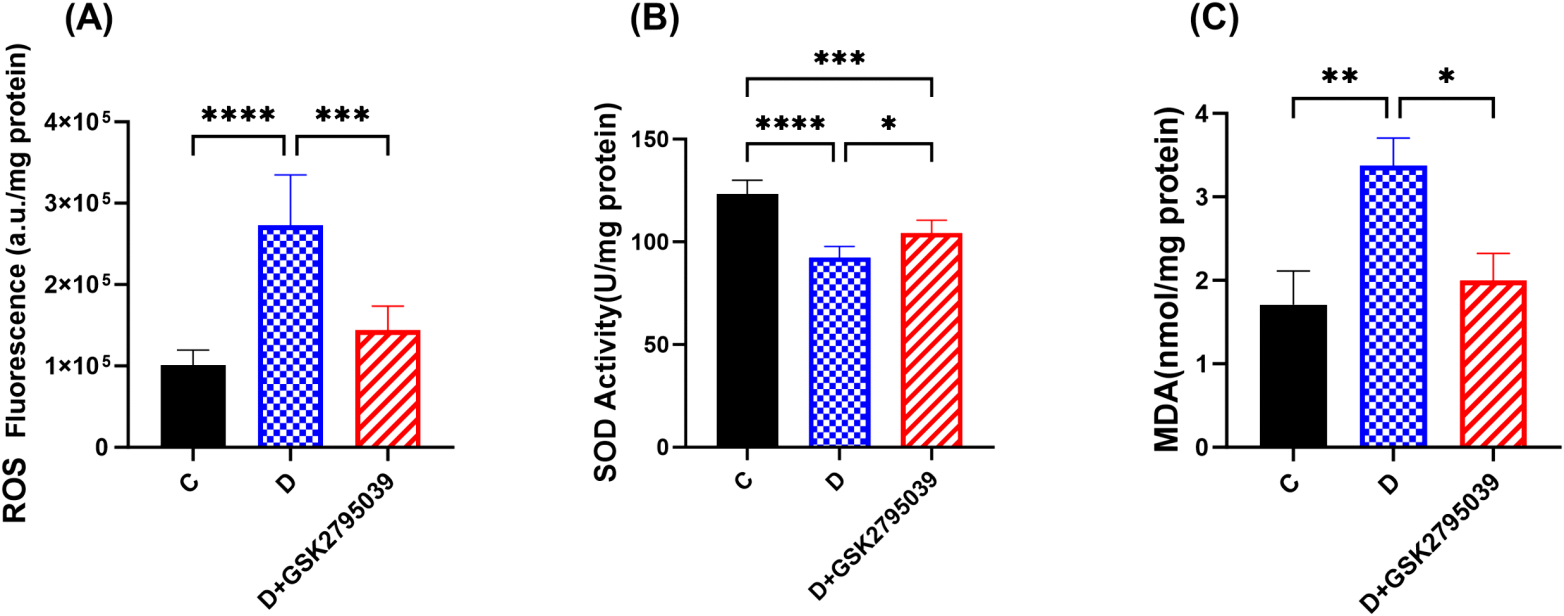
GSK2795039 pretreatment attenuates decompression-associated changes in oxidative stress indices in pulmonary arteries. Oxidative stress indices were assessed in pulmonary artery samples from Group C, Group D, and Group D + GSK2795039. (A) ROS production measured by DCFH-DA fluorescence intensity. (B) Superoxide dismutase (SOD) activity determined by the hydroxylamine method. (C) Malondialdehyde (MDA) levels determined by the thiobarbituric acid (TBA) assay. Data are presented as mean ± SD (n = 6 per group). Statistical analysis was performed using one-way ANOVA followed by Tukey’s post hoc test. * *p* < 0.05, ** *p* < 0.01, *** *p* < 0.001, **** *p* < 0.0001.

We next assessed oxidative stress in pulmonary arteries by measuring ROS production, SOD activity, and lipid peroxidation. Compared with Group C, decompression stress significantly increased ROS levels (Fig 2A; *p* < 0.0001), decreased SOD activity (Fig 2B; *p* < 0.0001), and elevated MDA levels (Fig 2C; *p* = 0.0029). Treatment with GSK2795039 attenuated these changes, decreasing ROS levels (*p* = 0.0002) and MDA levels (*p* = 0.0331) and partially restoring SOD activity (*p* = 0.0122) relative to Group D (Fig 2A–C). ROS and MDA levels did not differ between the Group C and Group D + GSK2795039 (*p* = 0.1966 and *p* > 0.9999, respectively), whereas SOD activity in Group D + GSK2795039 remained lower than that in Group C (*p* = 0.0003).

### GSK2795039 pretreatment attenuates decompression-associated changes in eNOS coupling-related readouts and increases BH4 and NOx levels in pulmonary arteries

**Fig 3:**
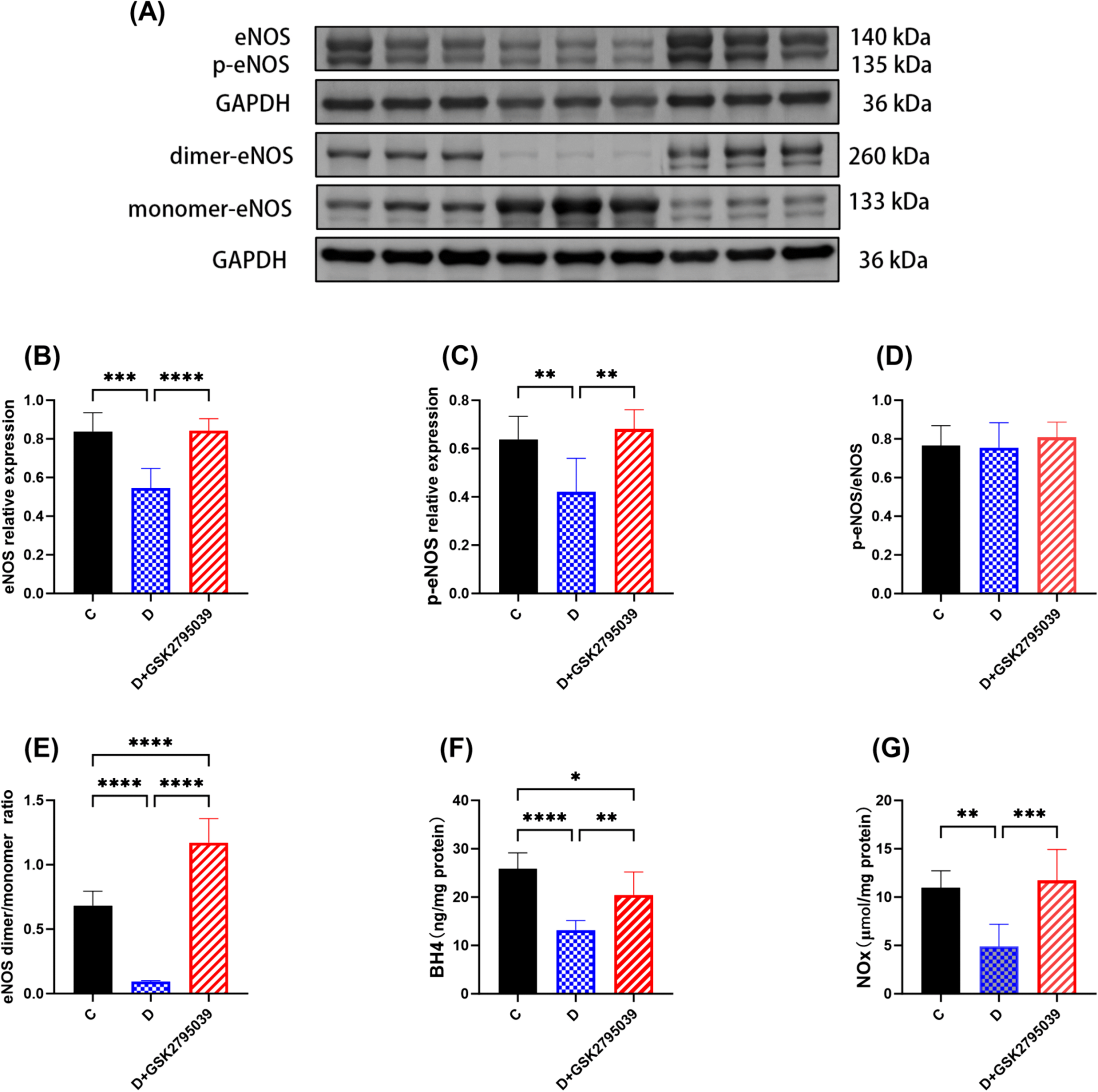
GSK2795039 pretreatment attenuates decompression-associated changes in eNOS coupling–related readouts and increases BH4 and NOx levels in pulmonary arteries. (A) Representative Western blots showing total eNOS, phospho-eNOS (Ser1177), eNOS dimer, eNOS monomer, and GAPDH in pulmonary artery homogenates from the Group C, Group D, and Group D + GSK2795039. For eNOS dimer/monomer analysis, samples were prepared and electrophoresed under non-reducing, non-boiled conditions. Separate GAPDH blots are shown for the reducing (eNOS, p-eNOS) and non-reducing (dimer/monomer) runs. (B) Densitometric quantification of total eNOS normalized to GAPDH. (C) Densitometric quantification of phospho-eNOS (Ser1177) normalized to GAPDH. (D) The p-eNOS/eNOS ratio. (E) The eNOS dimer/monomer ratio. (F) BH4 (tetrahydrobiopterin) content. (G) NOx (nitrate + nitrite) measured by the nitrate reductase method. Data are presented as mean ± SD (n = 6 per group). Statistical analysis was performed using one-way ANOVA followed by Tukey’s post hoc test. Exact group comparisons are indicated by brackets. * *p* < 0.05, ** *p* < 0.01, *** *p* < 0.001, **** *p* < 0.0001.

Decompression-induced alterations in eNOS signaling and the impact of GSK2795039 pretreatment were evaluated in pulmonary arteries by examining total eNOS expression, eNOS phosphorylation at Ser1177, and eNOS coupling-related parameters. Group D exhibited decreased total eNOS and phospho-eNOS (Ser1177) protein levels compared to the Group C (Fig 3A–C; *p* = 0.0001 and *p* = 0.0086, respectively), whereas the p-eNOS/eNOS ratio remained unaltered (Fig 3D; *p* = 0.9848). Group D showed reduced eNOS dimer levels and increased monomer levels under non-reducing conditions, resulting in a decreased dimer/monomer ratio compared with Group C (Fig 3A, E; *p* < 0.0001). BH4 content and NOx levels were also reduced after decompression (Fig 3F, G; *p* < 0.0001 and *p* = 0.0020, respectively). Pretreatment with GSK2795039 increased total eNOS (*p* < 0.0001), phospho-eNOS (Ser1177) (*p* = 0.0022), the eNOS dimer/monomer ratio (*p* < 0.0001), BH4 (*p* = 0.0075), and NOx (*p* = 0.0007) relative to Group D (Fig 3A–G). No significant differences were observed in total eNOS, phospho-eNOS, the p-eNOS/eNOS ratio, and NOx levels between Group C and Group D + GSK2795039 (*p* = 0.9969, *p* = 0.7784, *p* = 0.7682, and *p* = 0.8630, respectively). By contrast, the dimer/monomer ratio was higher (*p* < 0.0001) and BH4 levels were slightly increased (*p* = 0.0447) in Group D + GSK2795039 compared with Group C.

### GSK2795039 pretreatment attenuates decompression-associated increases in markers associated with endothelial activation in pulmonary artery homogenates

**Fig 4:**
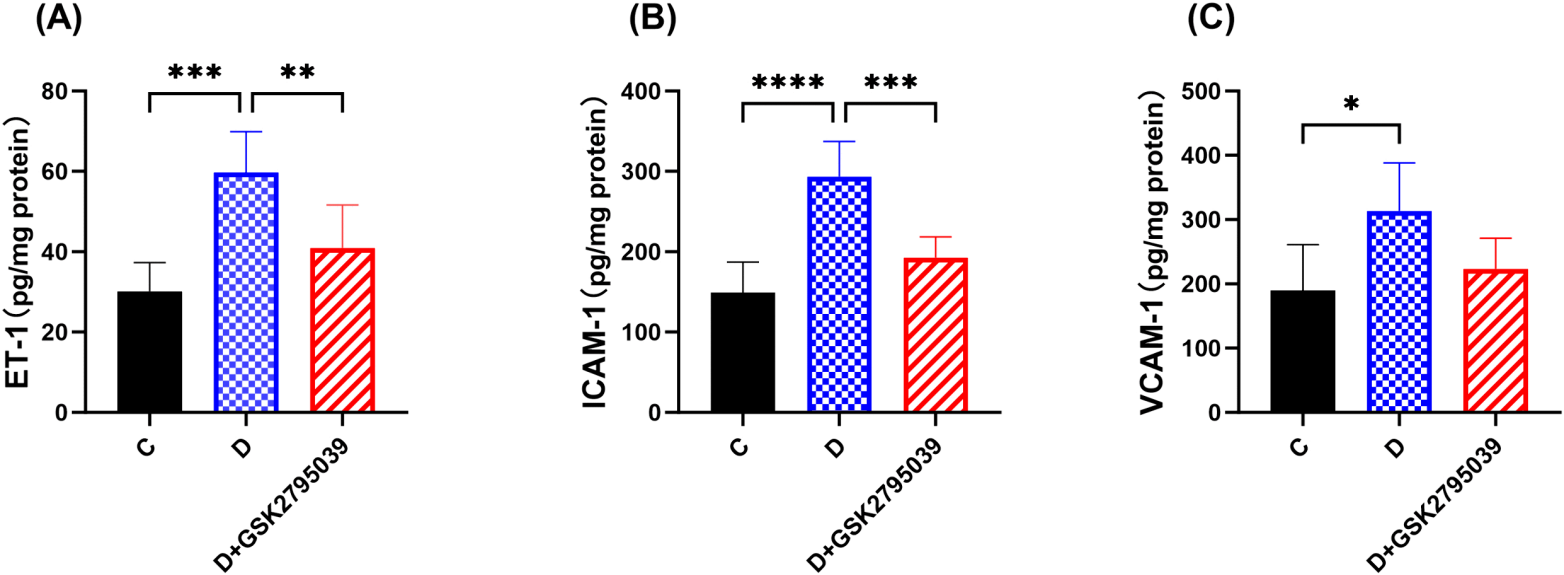
GSK2795039 pretreatment attenuates decompression-associated increases in markers associated with endothelial activation in pulmonary artery homogenates. ELISA quantification of (A) endothelin-1 (ET-1), (B) ICAM-1, and (C) VCAM-1 in pulmonary artery homogenates from Group C, Group D, and Group D + GSK2795039. Data are presented as mean ± SD (n = 6 per group). Statistical analysis was performed using one-way ANOVA followed by Tukey’s post hoc test. * *p* < 0.05, ** *p* < 0.01, *** *p* < 0.001, **** *p* < 0.0001.

To further characterize vessel-level changes indicative of endothelial activation post-decompression, we assessed ET-1, ICAM-1, and VCAM-1 levels in pulmonary artery homogenates using ELISA. Compared with Group C, Group D exhibited significantly elevated ET-1 (*p* = 0.0002), ICAM-1 (*p* < 0.0001), and VCAM-1 (*p* = 0.0137) levels (Fig 4A–C). Following pretreatment with GSK2795039, ET-1 and ICAM-1 levels decreased significantly compared to Group D (*p* = 0.0097 and *p* = 0.0007, respectively) and were no longer significantly different from Group C (*p* = 0.1525 and *p* = 0.1321, respectively), indicating attenuation of decompression-associated increases in these markers. VCAM-1 levels in Group D + GSK2795039 did not differ from Group C (*p* = 0.6511) and exhibited a decreasing tendency compared to Group D, although this difference did not reach statistical significance (*p* = 0.0768) (Fig 4A–C).

### GSK2795039 pretreatment partially improves endothelium-dependent relaxation in pulmonary arteries after decompression

**Fig 5:**
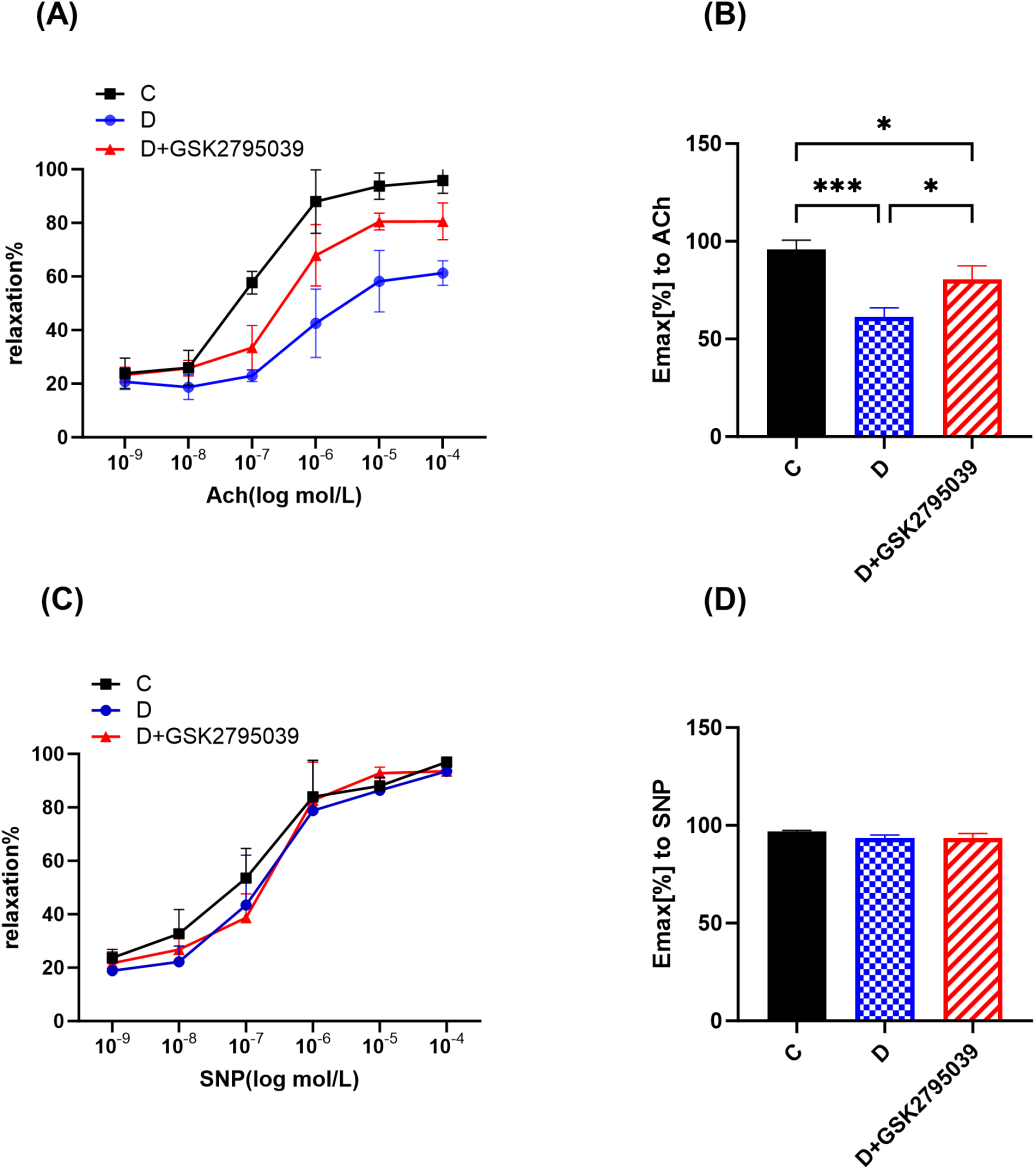
GSK2795039 pretreatment partially improves endothelium-dependent relaxation in pulmonary arteries after decompression. (A) Concentration-response curves for endothelium-dependent relaxation to acetylcholine (ACh, 10 ⁻ ⁹–10 ⁻ ⁴ M) in isolated pulmonary artery rings from Group C, Group D, and Group D + GSK2795039. (B) Maximal relaxation (Emax) to ACh. (C) Concentration-response curves for endothelium-independent relaxation to sodium nitroprusside (SNP, 10 ⁻ ⁹–10 ⁻ ⁴ M) in isolated pulmonary artery rings from Group C, Group D, and Group D + GSK2795039. (D) Maximal relaxation (Emax) to SNP. Relaxation is expressed as the percentage of phenylephrine (PE, 10 μM)-induced pre-contraction. Data are presented as mean ± SD (n = 3 per group). Statistical analysis was performed using one-way ANOVA (for Emax) or two-way repeated-measures ANOVA (for curves) followed by Bonferroni post hoc tests. * *p* < 0.05, ** *p* < 0.01, *** *p* < 0.001, **** *p* < 0.0001.

Finally, endothelial function was assessed using pulmonary artery ring assays. ACh (10 ⁻ ⁹–10 ⁻ ⁴ M)-evoked endothelium-dependent relaxation was significantly reduced in Group D compared with Group C, as illustrated in Fig 5A. Moreover, maximal relaxation (Emax) was lower in Group D (Fig 5B; *p* < 0.0001). GSK2795039 pretreatment improved ACh-evoked endothelium-dependent relaxation: Group D + GSK2795039 showed a leftward shift in the response curve compared with Group D (Fig 5A) and a partially restored Emax (Fig 5B; *p* = 0.0063 vs Group D). In contrast, there were no significant differences in endothelium-independent relaxation to SNP among the groups (Fig 5C), and SNP Emax remained similar across all groups (Fig 5D; *p* > 0.05).

## Discussion

This study demonstrates that simulated air diving is associated with pulmonary vascular endothelial dysfunction, accompanied by increased NOX2- associated oxidative stress and alterations in eNOS coupling–related parameters. Decompression stress was associated with increased NOX2 expression and elevated oxidative stress markers in rat pulmonary arteries, along with a lower eNOS dimer/monomer ratio and reduced nitric oxide metabolites (NOx). Pharmacological inhibition of NOX2 with GSK2795039 attenuated these oxidative changes and partially enhanced endothelium-dependent vasodilation. Collectively, these findings support the hypothesis that the NOX2-associated oxidative stress–eNOS coupling axis may contribute to decompression-induced vascular damage.

Oxidative stress has been implicated in the pathophysiology of decompression illness and diving-related vascular injury, although the dominant enzymatic sources of ROS may differ depending on the experimental model and exposure conditions [22,23]. Both experimental and clinical studies have demonstrated elevations in oxidative and inflammatory markers following decompression, including in models of decompression-associated lung injury [18,24].

The dominant ROS-generating pathways, including mitochondrial and NADPH oxidase–derived sources, may differ across experimental settings, complicating mechanistic attribution to a single enzymatic source [25]. Recent studies have implicated NOX2-dependent oxidative signaling in decompression-associated pulmonary injury and endothelial damage [26]. Consistent with these reports, our data demonstrated upregulation of NOX2 protein expression in pulmonary arteries after decompression, accompanied by elevated ROS levels and reduced SOD activity. Pharmacological inhibition of NOX2 with GSK2795039, a selective small-molecule inhibitor [27,28], attenuated these oxidative stress markers, supporting a role for NOX2 in decompression-induced ROS generation. Nevertheless, the oxidative stress indices employed in this study reflect overall oxidative burden and do not directly identify the enzymatic source(s) of ROS; contributions from other NADPH oxidase isoforms or mitochondrial pathways cannot be excluded [25,26]. Furthermore, although GSK2795039 has been characterized as a NOX2-selective compound [27,28], the possibility of off-target antioxidant effects should be considered when interpreting in vivo pharmacological data [25].

A key mechanistic consideration is that oxidative stress can promote BH4 oxidation, predisposing eNOS to uncoupling and reducing NO bioavailability [29]. In vascular diseases, such as hypertension, excess superoxide oxidizes BH4 and promotes eNOS uncoupling [9]. Consistent with this framework, decompression stress was associated with reduced BH4 levels and a lower eNOS dimer/monomer ratio, both of which were improved by NOX2 inhibition [27]. Because uncoupled eNOS can produce superoxide rather than NO, eNOS uncoupling may amplify oxidative stress and further limit NO bioavailability [10,30]. The partial restoration of the dimer/monomer ratio with GSK2795039 suggests NOX2-associated oxidative stress may contribute to eNOS uncoupling, thereby perpetuating a cycle of oxidative stress and endothelial dysfunction. However, the diminished BH4 levels, reduced eNOS dimer/monomer ratio, and lower NOx levels should be viewed as indirect markers of altered eNOS coupling rather than conclusive evidence of uncoupled eNOS activity in this model.

To link these biochemical alterations to vascular function, we used wire myography to evaluate pulmonary artery reactivity [30], addressing a limitation of some previous diving studies in which functional assays were not performed [19]. Decompression stress resulted in impaired ACh-induced endothelium-dependent relaxation. In contrast, responses to the endothelium-independent vasodilator SNP remained intact, suggesting that the impairment aligns with an endothelial factor rather than primary vascular smooth muscle dysfunction [16]. The improvement in ACh-mediated relaxation with GSK2795039 provides functional support for NOX2 inhibition–sensitive mechanisms in decompression-induced vascular dysfunction. Nevertheless, due to the limited number of animals involved in the vascular reactivity experiments, these functional findings should be interpreted with caution rather than as conclusive evidence.

Endothelial dysfunction in DCS is commonly linked to inflammatory activation [22,26]. We observed increased levels of ET-1, ICAM-1, and VCAM-1 after decompression, consistent with previous findings indicating rises in vasoactive and endothelial activation markers after decompression exposure [16,19,31]. The rise in adhesion molecules indicates endothelial activation, a process that may precede leukocyte recruitment in DCS [26,32]. Treatment with GSK2795039 attenuated these markers, suggesting that NOX2-related oxidative stress may contribute to inflammatory and vasoconstrictive signaling. Because these proteins were measured in whole pulmonary artery homogenates, their interpretation should reflect vessel-level alterations indicative of endothelial activation. Further research is needed to elucidate the specific pathways connecting ROS to these markers.

**Limitations of the study.** This study has several limitations. First, most molecular and biochemical endpoints were obtained from whole pulmonary artery homogenates rather than endothelial cell–specific samples; therefore, these results should be interpreted as vessel-level readouts, and contributions from non-endothelial vascular cells cannot be entirely excluded. Second, pharmacological inhibition with GSK2795039 may involve off-target effects; future studies using genetically modified models (e.g., NOX2/Cybb knockout rats and/or endothelial-specific knockout rats) would strengthen causal inference. Third, although BH4 was quantified, future work should assess BH4 redox status (e.g., the BH4/BH2 ratio) using more specific analytical methods (e.g., HPLC or LC–MS) to better characterize BH4 oxidation and eNOS uncoupling. Fourth, the direct quantification of eNOS-derived superoxide (e.g., L-NAME–inhibitable O^2−^) was not conducted, nor were cell-type–resolved analyses performed, which hinders the ability to attribute the observed oxidative stress indicators and coupling alterations mechanistically. Finally, the present model represents an acute response to a single simulated dive, and chronic adaptations may differ.

## Conclusions

In conclusion, simulated air diving was associated with pulmonary vascular endothelial dysfunction, as reflected by impaired vasodilation and markers of endothelial activation measured at the vessel level. Our data suggest that NOX2-associated oxidative stress may contribute to these changes, potentially in conjunction with reduced BH4 availability, an altered eNOS coupling balance (lower dimer/monomer ratio), and decreased nitric oxide metabolites. In our experimental setting, pharmacological inhibition of NOX2 attenuated these molecular indices and was accompanied by improved endothelial function. Together, these findings indicate that targeting NOX2-associated oxidative stress and supporting eNOS coupling could be explored as a strategy to reduce decompression-associated vascular injury, pending further validation across models and conditions, and with additional studies incorporating more direct assessments of ROS sources and eNOS-derived superoxide/BH4 redox status.

## Supporting information

S1 File Data. (XLSX)

S2 File Raw image. (PDF)

S3 File S3 Reagents, Kits, and Antibodies. (XLSX)
