# Supplementary figures and images for: NOX2 inhibition attenuates oxidative stress and eNOS uncoupling in pulmonary arteries of rats following simulated air diving
Source: PLoS One. 2026 Jul 2;21(7):e0351145. doi: 10.1371/journal.pone.0351145 (PMC13327252; doi:10.1371/journal.pone.0351145)

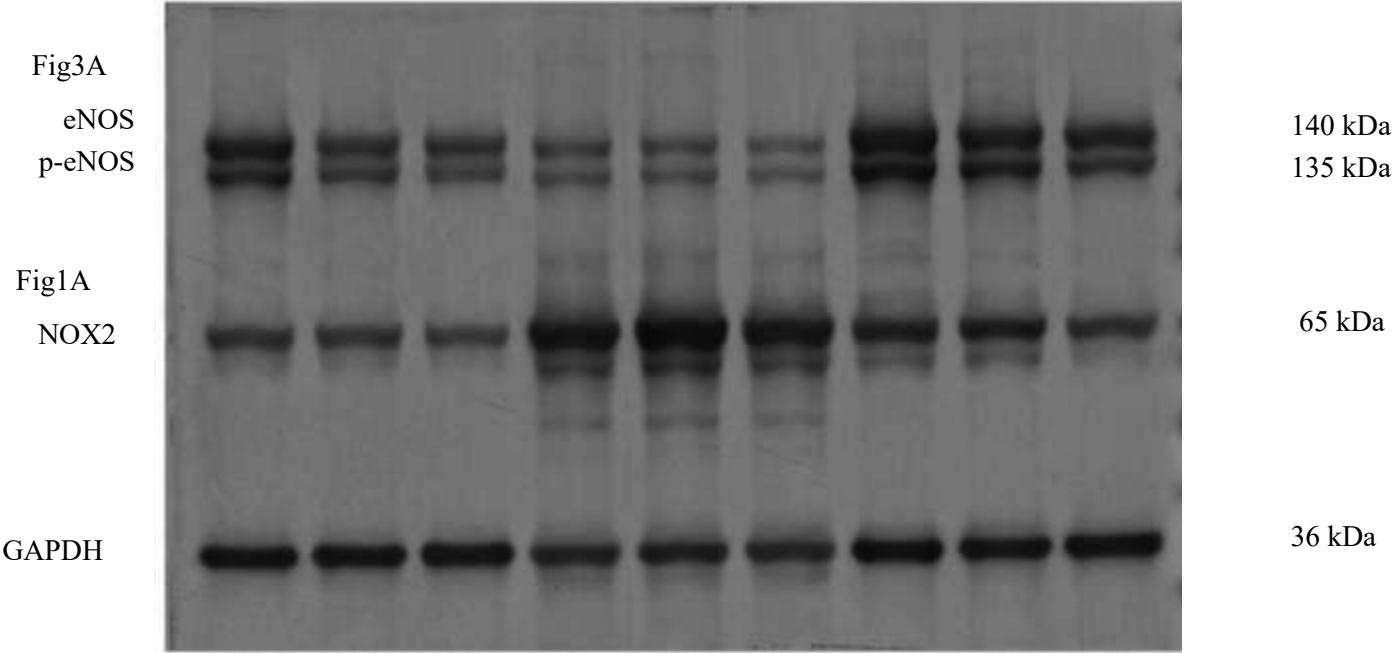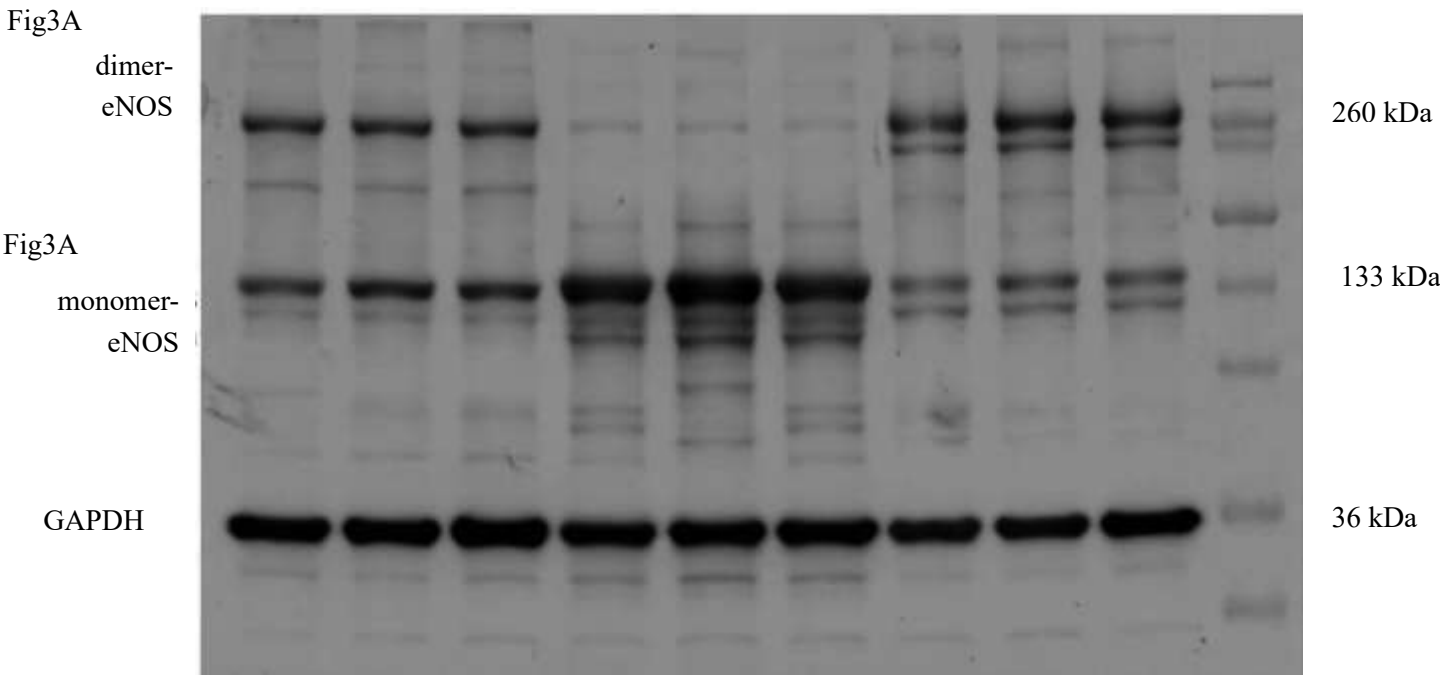

Supplement: S2 File — (PDF) [file pone.0351145.s002.pdf]
