# Supplementary material for: NOX2 inhibition attenuates oxidative stress and eNOS uncoupling in pulmonary arteries of rats following simulated air diving
Source: PLoS One. 2026 Jul 2;21(7):e0351145. doi: 10.1371/journal.pone.0351145 (PMC13327252; doi:10.1371/journal.pone.0351145)
Supplement: Attachment — Submitted filename: Response to Reviewers.docx [file pone.0351145.s005.docx]

Response Letter to PLOS ONE Submission

Paper ID: PONE-D-25-62136

Paper Title: NOX2/ROS-mediated eNOS uncoupling leads to endothelial dysfunction in the pulmonary arteries of simulated air diving rats

Dear Academic Editor (Dr. Zhang) and the reviewers:

Thank you for the opportunity to revise our manuscript. We sincerely thank the editor and all reviewers for their valuable feedback that we have used to improve the quality of our manuscript.

During the major revision, Reviewer 1 raised an important methodological concern regarding the limited specificity of the inhibitor used in the original submission. To directly address this key issue and to improve the reliability and interpretability of our conclusions, we replaced the inhibitor in the third group with GSK2795039 (the reagent information and dosing regimen have been updated in the Materials and methods section).

Given that the key intervention factor was changed, to avoid potential confounding introduced by different animal batches and/or experimental time periods and to ensure comparability among the three groups, we conducted a complete repeat of the experiments using an independent new animal cohort for the control, decompression, and inhibitor-treated groups, and remeasured all original endpoints. In addition, following the reviewers’ suggestions, we added/supplemented measurements of ICAM-1, VCAM-1, BH4,SOD, and MDA, as well as functional assays using pulmonary artery rings, to strengthen the mechanistic and functional evidence.

All data presented in the revised manuscript are derived from the repeated experiments in this new cohort; the corresponding figures, statistical analyses, and supplementary materials have been updated accordingly. All additional experiments were performed within the scope of the original ethical approval.

We submitted 3 files, including a revised manuscript, a track changes file (to highlight differences between the revised and the original manuscript), and this file, i.e., the response letter. In the following, we respond to each of your concerns and recommendations point-by-point. All changes are shown in the tracked-changes file, and corresponding locations are provided for the clean manuscript.

We are hopeful that the revisions we have made satisfy the concerns raised by the reviewers and look forward to your positive response.

Sincerely,

Wang Song

Exercise Immunology Center, Wuhan Sports University
Wuhan, China

Response to the Editor #

*Journal Requirement 1 (Style requirements and file naming)*

*Comment#1. Please ensure that your manuscript meets PLOS ONE's style requirements, including those for file naming.*

Response:

We have revised the manuscript to comply with PLOS ONE style and formatting requirements (including section structure, figure legends, and reference formatting) and ensured that all files follow the requested naming conventions. In the resubmission package, we provide separate files for (i) the point-by-point response, (ii) the tracked-changes manuscript, and (iii) the clean revised manuscript. Figures are provided as separate high-resolution TIFF files, and Supporting Information files are provided separately as required.

Files submitted (named as requested):

- Response to Reviewers: Response to Reviewers
- Revised Manuscript with Track Changes: Revised Manuscript with Track Changes(docx)
- Clean Manuscript: Manuscript (PDF)
- Cover letter: Cover letter (Revised) (.docx)
- Figure files: Fig1, Fig2, Fig3, Fig4, Fig5 (TIFF)
- Supporting Information: S1 Data(xlsx); S2 Raw image (PDF); S3 Reagents, Kits, and Antibodies(xlsx)

*Journal Requirement 2 (Animal experiments details in Methods)*

*Comment#2. To comply with PLOS ONE submissions requirements, in your Materials and methods section, please provide additional information regarding the experiments involving animals and ensure you have included details on (1) methods of sacrifice, (2) methods of anesthesia and/or analgesia, and (3) efforts to alleviate suffering.*

Response：

Thank you for your comment. We have revised the Materials and methods section to provide the requested animal welfare and procedural details.

(1) Methods of sacrifice/euthanasia:

Rats were euthanized while under deep anesthesia by exsanguination via cardiac puncture followed by thoracotomy. Death was confirmed by cessation of heartbeat and respiration.

(2) Anesthesia/analgesia:

Before tissue collection, rats were deeply anesthetized with sodium pentobarbital (50 mg/kg, i.p., with supplemental doses as needed). Adequate depth of anesthesia was confirmed by the absence of pedal withdrawal and corneal reflexes. We also specified the predefined rescue analgesia plan: buprenorphine (0.05 mg/kg, subcutaneously) would have been administered if persistent pain/distress had occurred; however, no animal reached a humane endpoint and buprenorphine was not administered in this study.

(3) Efforts to alleviate suffering:

Animals were acclimatized for at least one week prior to experimentation and were monitored daily for general appearance, body weight, grooming behavior, and respiratory pattern. During and after the simulated diving procedure, rats were closely observed for signs of discomfort or neurological symptoms (e.g., labored breathing, tremor, paralysis, or prolonged recumbency). Predefined humane endpoints and immediate euthanasia under deep anesthesia were in place if persistent distress had been observed.

Changes in the clean manuscript:

- Materials and methods (Ethics statement): [Page 4-5, Lines 42–66]

*Journal Requirement 3 (Use the PLOS LaTeX template)*

*Comment#3. Please update your submission to use the PLOS LaTeX template.*

Response:

Thank you for the instruction. We have converted the revised manuscript to the PLOS LaTeX template and are providing the compiled PDF, as part of the resubmission. Figures are uploaded separately as high-resolution TIFF files in accordance with PLOS ONE requirements.

Files provided:

- Compiled PDF: Manuscript (LaTeX PDF)

*Journal Requirement 4 (Funding information placement and Funding Statement update)*

*Comment#4.Remove any funding-related text from the manuscript and indicate how you would like to update the Funding Statement; include amended statements in the cover letter.*

Response:

We have removed all funding-related text from the manuscript (including the “Funding” section) and confirm that funding information should appear only in the online Funding Statement.

Changes in the Requested Funding Statement:

- “The authors received no specific funding for this work.”

Changes in the Cover letter:

- We have included the amended Funding Statement exactly as written above.

*Journal Requirement 5 (Minimal data set / Data Availability confirmation)*

*Comment#5. We note that your Data Availability Statement is currently as follows: “All relevant data are within the manuscript and its Supporting Information files.”*

*Please confirm at this time whether or not your submission contains all raw data required to replicate the results of your study. Authors must share the “minimal data set” for their submission.*

Response：

We confirm that the minimal data set underlying the reported findings is fully available. All underlying/raw values required to reproduce the analyses and figures are provided as Supporting Information.

Specifically, S1 Data (xlsx) contains the underlying values for Fig 1–Fig 5, including the raw/underlying measurements used for summary statistics, the data used to generate graphs, and the quantitative values underlying any image-based analyses. These data enable replication of the reported statistical analyses and results.

Updated Data Availability Statement:

All relevant data are within the manuscript and its Supporting Information files. The underlying data for Fig 1–Fig 5 are provided in S1 Data (xlsx), blot images are provided in S2 Raw image (PDF).

Changes in the clean manuscript:

- Supporting Information: S1 Data and S2 Raw image.

*Journal Requirement 6 (Original uncropped and unadjusted blot/gel images)*

*Comment#6. PLOS ONE now requires that authors provide the original uncropped and unadjusted images underlying all blot or gel results reported in a submission’s figures or Supporting Information files.*

*In your cover letter, please note whether your blot/gel image data are in Supporting Information.*

Response：

We have provided the original uncropped and unadjusted images underlying all blot results (Fig 1 and Fig 3) reported in the manuscript. The raw images corresponding to the blots in Fig1 and Fig3 are included in the Supporting Information file S2 Raw image.

Changes in the Cover letter:

- We noted that the original uncropped and unadjusted blot images are provided in Supporting Information (S2 Raw image).

*Journal Requirement 7 (“data not shown”)*

*Comment#7. “data not shown” is not permitted; provide accessible data, add a citation, or remove the phrase if not core.*

Response：

Thank you for noting this. We have removed the phrase “data not shown” from the manuscript. All data underlying the findings reported in this study are provided in the submission as Supporting Information (raw data files; see S1 Data), and no references to inaccessible data remain.

*Journal Requirement 8 (Ethics statement only in Methods)*

*Comment#8. Your ethics statement should only appear in the Methods section of your manuscript. If your ethics statement is written in any section besides the Methods, please delete it from any other section.*

Response：

We confirm that the ethics statement now appears only in the Materials and methods section. Any duplicate ethics statements that previously appeared outside the Materials and methods section have been removed to comply with PLOS ONE requirements.

*Journal Requirement 9 (Remove figures from within manuscript file)*

*Comment#9. Please remove your figures from within your manuscript file, leaving only the individual TIFF/EPS image files, uploaded separately. These will be automatically included in the reviewers’ PDF.*

Response：

We have removed all embedded figures from the manuscript file. All figures are provided as separate figure files (Fig1–Fig5) uploaded individually, while the manuscript contains only the figure callouts and figure legends.

*Journal Requirement 10 (Reviewer-suggested citations)*

*Comment#10. If the reviewer comments include a recommendation to cite specific previously published works, please review and evaluate these publications to determine whether they are relevant and should be cited. There is no requirement to cite these works unless the editor has indicated otherwise.*

Response:

In response to the reviewer’s concern about using whole-vessel tissue measurements (ROS, NO, and ET-1) rather than isolated endothelium or primary endothelial cells, we recognized that additional supporting references would strengthen the methodological justification. We therefore added relevant citations in the Methods to support the use of whole-vessel readouts in related decompression/vascular injury contexts, and we clarified the rationale as well as the limitation that these measurements are not endothelium-specific. We also tempered interpretation accordingly in the Results/Discussion.

Changes in the clean manuscript:

- Materials and methods (Tissue collection): [Page 3, Lines 91–111]

Response to Reviewer #1

*Comment#1. Novelty is limited and not convincingly demonstrated.*

*Although the authors attempt to connect NOX2-derived ROS with eNOS uncoupling during simulated diving, both pathways are already well described, and the manuscript does not provide compelling evidence that the findings represent a novel mechanism specific to decompression-related vascular injury.*

Response:

We thank the reviewer for the comment regarding novelty. We agree that NOX2-derived ROS signaling and BH4-dependent eNOS uncoupling are well established in chronic vascular disease. Our study does not claim novelty of these pathways per se; rather, it tests in an acute simulated air-diving/decompression model whether a defined enzymatic ROS source (NOX2) contributes to BH4 loss/eNOS uncoupling and pulmonary vascular dysfunction.

To address this concern, we revised the Introduction and Discussion to more clearly define the knowledge gap in the decompression setting and to avoid overstating novelty. Specifically, we now emphasize that prior diving/decompression studies often relied on systemic oxidative indices or nonspecific antioxidant interventions, whereas source-specific evidence linking NOX2 activation to BH4 depletion, eNOS uncoupling, reduced NO bioavailability, and functional impairment in pulmonary arteries has been limited. We also tempered language throughout the Abstract/Conclusion (e.g., replacing “central role/first evidence” with “evidence consistent with a NOX2-dependent contribution”).

Experimentally, the revised study integrates biochemical endpoints (NOX2, ROS/oxidative indices, BH4, NO metabolites, and eNOS coupling status) with wire-myography assessment of endothelium-dependent relaxation and includes pharmacologic NOX2 inhibition (GSK2795039). The concordant improvements in molecular and functional readouts with NOX2 inhibition support a NOX2-dependent contribution to post-decompression endothelial dysfunction in this model.

Changes in the clean manuscript:

- Introduction: revised to clarify the decompression-setting knowledge gap and avoid novelty overstatements. [Page 1-2, Lines 1-40]
- Discussion: revised opening and framing to align with the decompression model and temper claims. [Page 9-11, Lines 361-431]
- Abstract/Conclusion: revised to use cautious language consistent with the evidence. [Page 1; Page 11, Lines 432–442]

*Comment#2. Mechanistic evidence is insufficient.*

*The study presents only associative data linking NOX2, ROS, and eNOS uncoupling. No deeper causal validation is provided (e.g., NOX2 knockout models, genetic or pharmacologic rescue beyond apocynin). The reliance on apocynin—an agent with broad antioxidant effects and limited NOX2 specificity—weakens the mechanistic conclusions.*

*Additionally, the manuscript lacks functional vascular assays (e.g., endothelium-dependent vasodilation tests), which are essential to substantiate the claim of “endothelial dysfunction.”*

Response:

We thank the reviewer for this important critique and agree that the original submission was limited by (i) reliance on apocynin, which has limited NOX2 specificity, and (ii) the lack of functional vascular assays to support “endothelial dysfunction.”

To address these concerns, we substantially strengthened the revision as follows:

1. Improved NOX2 specificity: We replaced apocynin with the NOX2 inhibitor GSK2795039 administered prior to pressurization (supported by prior studies for NOX2 inhibition; Hirano et al., 2015; Oliveira et al., 2022) and updated all Methods/Results accordingly. NOX2 inhibition attenuated decompression-induced oxidative stress and improved NO-related readouts.

Changes in the clean manuscript:

- Materials and methods (Experimental grouping): [Page 3, Lines 67-81]
- Results (Fig1-Fig5): [Page 7-9, Lines 252-360]
- Discussion: [Page 19, Lines 361-431]

2. Mechanistically informative intermediate (BH4): We added direct quantification of tetrahydrobiopterin (BH4) (Fig 3F), a key cofactor whose depletion is consistent with eNOS uncoupling, providing a mechanistically relevant link between oxidative stress and eNOS coupling/NO signaling.

Changes in the clean manuscript:

- Introduction: [Page 2, Lines 16-26]
- Materials and methods (ELISA assays): [Page 6, Lines222-239]
- Results (Fig 3F): [Page 9, Lines 297-304]
- Discussion: [Page 10, Lines 391-401]

3. Functional vascular validation: We added wire-myography vascular reactivity assays, including endothelium-dependent vasodilation (ACh) and endothelium-independent vasodilation (SNP) (Fig 5A–D), providing direct functional evidence of endothelial dysfunction and helping distinguish endothelial impairment from altered smooth muscle responsiveness.

Changes in the clean manuscript:

- Materials and methods (Assessment of vascular endothelial function): [Page 6, Lines 209-221]
- Results (Fig 5A–D): [Page 9, Lines 338-360]
- Discussion: [Page 10, Lines 402-410]

4. Expanded endothelial phenotype markers: We added ICAM-1 and VCAM-1 as established markers of endothelial activation/inflammation (Fig 4B–C), supporting the biological relevance of the observed endothelial injury phenotype.

Changes in the clean manuscript:

- Materials and methods (ELISA assays): [Page 6, Lines222-239]
- Results (Fig 4B–C): [Page 8-9, Lines 320-337]
- Discussion: [Page 10, Lines 411-419]

Across these added endpoints, GSK2795039 produced concordant improvements (reduced oxidative indices, preservation of BH4, improved NO-related measures, improved ACh-mediated relaxation, and attenuation of ICAM-1/VCAM-1), supporting a NOX2-dependent contribution to post-decompression endothelial dysfunction in this model. We acknowledge that genetic loss-of-function approaches (e.g., NOX2 knockout) would provide additional orthogonal validation; therefore, we also tempered mechanistic language in the Abstract/Discussion to align with the strength of the current evidence.

*Comment#3. Concerns regarding assay methodology.*

*The ROS, NO, and ET-1 assays are performed on whole vessel tissue rather than isolated endothelium or primary endothelial cells. If this approach represents an accepted standard for the diving-related vascular field, appropriate references must be provided. Otherwise, the measurements may not accurately reflect endothelial-specific changes.*

Response:

We thank the reviewer for raising this important methodological concern. We agree that biochemical assays performed on whole-vessel pulmonary artery preparations cannot be interpreted as strictly endothelium-exclusive. In the revised manuscript, we therefore (i) explicitly describe ROS/NO/ET-1 and oxidative stress indices measured from pulmonary artery homogenates/cell preparations as integrated vascular wall/tissue-level readouts, (ii) add field-appropriate references supporting the common use and known limitations of intact-vessel/tissue approaches in vascular injury/decompression research, and (iii) ensure that conclusions regarding “endothelial dysfunction” are anchored primarily in endothelium-specific functional evidence rather than in bulk oxidative indices alone.

Specifically, in the Methods (Tissue collection), we now clarify that whole-vessel measurements reflect mixed-cell contributions and we cite relevant decompression-related vascular studies that combine vessel-level biomarkers with ex vivo vascular reactivity assays. In addition, we added endothelium-dependent and endothelium-independent vasodilation testing (wire myography; Fig 5A–D) as direct functional validation of endothelial impairment, and we expanded endothelial phenotype validation by including ICAM-1/VCAM-1 (Fig 4B–C) together with mechanistically relevant NO-signaling endpoints (e.g., BH4; Fig 3F).

Finally, we revised the Discussion/Limitations to avoid language implying endothelial exclusivity for whole-vessel biochemical assays and to state explicitly that such assays reflect vascular wall/tissue changes, while endothelial dysfunction conclusions are supported by functional testing and endothelial-enriched molecular markers.

Changes in the clean manuscript:

- Materials and methods (Tissue collection): clarified that ROS/NO/ET-1/SOD/MDA assays were performed on pulmonary artery whole-vessel/tissue-derived preparations and added supporting references for the intact-vessel/tissue approach in decompression-related vascular research. [Page 3-4, Lines 91-111]
- Materials and methods (Biochemical assays): clarified that ROS /NO /SOD/ MDA assays were performed on pulmonary artery whole-vessel/tissue-derived preparations and added supporting references for the intact-vessel/tissue approach in decompression-related vascular research. [Page 5-6, Lines 162-208]
- Materials and methods (ELISA assays): added ICAM-1 and VCAM-1 as established markers of endothelial activation/inflammation, supporting the biological relevance of the observed endothelial injury phenotype [Page 6, Lines 222-239]
- Materials and methods (Assessment of vascular endothelial function): added vascular reactivity assays [Page 12-13, Lines 218-230]
- Results: added ICAM-1/VCAM-1 (Fig 4B–C) and BH4 (Fig 3F) to strengthen endothelial phenotype/mechanistic support. [Page 16-18, Lines 296-345]
- Results: added vascular reactivity assays (endothelium-dependent and -independent vasodilation; Fig 5A–D). [Page 6, Lines 209-221]
- Discussion/Limitations: tempered interpretation to avoid endothelium-exclusive claims for whole-vessel assays; emphasized functional anchoring. [Page 9-11, Lines 361-431]

References added:

- Ref #16: Zhang K, Wang D, Jiang Z, Ning X, Buzzacott P, Xu W. Endothelial dysfunction correlates with decompression bubbles in rats. Sci Rep. 2016; 6:33390. Epub 20160912. doi: 10.1038/srep33390.
- Ref #17: Berenji Ardestani S, Matchkov VV, Hansen K, Jespersen NR, Pedersen M, Eftedal I. Extensive Simulated Diving Aggravates Endothelial Dysfunction in Male Pro-atherosclerotic ApoE Knockout Rats. Front Physiol. 2020; 11:611208. Epub 20201223. doi: 10.3389/fphys.2020.611208.
- Ref #18: Lu C, Gu D, Chen H, Chen L, Chen J, Weng Y, et al. BM-MSCs mitigate lung injury in a rat model of decompression sickness. PLOS ONE. 2025;20(8): e0326618. Epub 20250821. doi: 10.1371/journal.pone.0326618.
- Ref #19: Zhang K, Wang M, Wang H, Liu Y, Buzzacott P, Xu W. Time Course of Endothelial Dysfunction Induced by Decompression Bubbles in Rats. Front Physiol. 2017; 8:181. Epub 20170323. doi: 10.3389/fphys.2017.00181.
- Ref #20: Zanetti M, d'Uscio LV, Peterson TE, Katusic ZS, O'Brien T. Analysis of superoxide anion production in tissue. Methods Mol Med. 2005;108:65-72. doi: 10.1385/1-59259-850-1:065.

*Comment#4. The Discussion requires major restructuring.*

*The writing contains redundancies, abrupt shifts between topics, and several unclear explanations. The lack of logical flow significantly diminishes the readability and interpretability of the findings.*

*Given these scientific and editorial issues, the manuscript requires major revision and additional mechanistic data before it can be suitable for publication.*

Response:

We appreciate the reviewer’s candid assessment and agree that the original Discussion contained redundancies and an insufficiently clear logical flow. We have therefore substantially rewritten and reorganized the Discussion to improve readability, strengthen internal transitions, and align interpretation with the revised dataset.

Editorial restructuring: The revised Discussion now follows a clearer sequence: (1) summary of principal findings and study scope; (2) NOX2 as an oxidant source in the decompression model; (3) evidence consistent with BH4 loss/eNOS uncoupling and reduced NO signaling; (4) functional validation using vascular reactivity assays; (5) endothelial activation/inflammatory phenotype; and (6) limitations and future directions. Redundant text was removed and overlapping content consolidated to avoid abrupt topic shifts.

Scientific strengthening: To address the request for additional mechanistic/functional support, we replaced apocynin with the more selective NOX2 inhibitor GSK2795039, added BH4 quantification (Fig 3F), added endothelium-dependent and endothelium-independent vasodilation testing (Fig 5A–D), and added ICAM-1/VCAM-1 as endothelial activation markers (Fig 4B–C). These additions enable a more coherent, evidence-aligned Discussion linking molecular changes to functional outcomes without overstating causality.

Finally, we tempered language throughout to ensure conclusions match the strength of the current evidence.

Changes in the clean manuscript:

- Discussion: major reorganization and rewriting to remove redundancy, improve transitions, and establish a clearer mechanism-to-function flow. [Page 9-11, Lines 361-431]
- Results/Discussion: integrated newly added data (GSK2795039; BH4 Fig 3F; ICAM-1/VCAM-1 Fig 4B–C; vascular reactivity Fig 5A–D) into the revised narrative. [Page 8-9, Lines 305-360; Page 9-11, Lines 361-431]
- Abstract /Discussion/Conclusion: revised wording to avoid overstatement and reflect evidence strength. [Page 1-2; Page 19-22, Lines 367-439; Page 22-23, Lines 440-450]

Response to Reviewer #2

*Comment#1. In the simulated diving procedure, mention the normal pressure range of the* *pressurized chamber in which control animals were housed.*

Response:

Thank you for this helpful suggestion. We have revised the Materials and methods section to clearly specify the chamber pressure condition for the control animals. Specifically, we state that control rats were housed in the same hyperbaric chamber at normobaric pressure (100 kPa, 1 ATA) for an equivalent duration, without pressurization or decompression.

Changes in the clean manuscript:

- Materials and methods (Experimental grouping / Simulated diving procedure): [Page 3, Lines 67-90]

*Comment#2. Specify the rat strain used for the study.*

Response:

Thank you for pointing this out. We have revised the Materials and methods section to explicitly specify the rat strain used in this study. We now state that male Sprague–Dawley rats (280–320 g) were used.

Changes in the clean manuscript:

- Materials and methods (Ethics statement): [Page 2-3, Lines 41-66]

*Comment#3. Intraperitoneal injection of apocynin: was it just a single dose before subjecting to diving procedure.*

Response:

Thank you for the comment. In the revised study, apocynin was removed and replaced with the NOX2 inhibitor GSK2795039 to address concerns about apocynin’s limited specificity. We have clarified in the Materials and methods that GSK2795039 was administered as a single intraperitoneal dose (100 mg/kg) 30 min before pressurization (i.e., one dose prior to the simulated diving/decompression procedure).

Changes in the clean manuscript:

- Materials and methods (Experimental grouping): [Page 3, Lines 67-81]

*Comment#4. Elaborate on the behavioral indicators observed after the simulated diving procedure other than analysis done using pulmonary artery tissue sample. How long after the procedure was this done and also include details on pulmonary artery tissue collection and the time point. How many tissues have been used for each of the analysis performed?*

Response:

Thank you for this important suggestion. We have revised the Materials and Methods to specify (i) post-dive behavioral/clinical observations, (ii) the timing and procedure of pulmonary artery collection, and (iii) the number of animals/tissues used for each analysis.

1. Post-procedure monitoring (timing and indicators):

We state that animals were monitored continuously from chamber exit until euthanasia/tissue harvest (within 60 min after decompression) for general condition and decompression-related signs, including activity level, grooming, dyspnea/respiratory distress, lethargy, abnormal gait/ataxia, hindlimb weakness/paralysis, and convulsions. No formal scoring system was applied; observations were recorded qualitatively (presence/absence of overt abnormal signs).

Changes in the clean manuscript:

- Materials and methods (Simulated diving procedure): [Page 3, Lines 82-90]

2. Pulmonary artery collection (time point and details):

We clarify that pulmonary arteries were harvested 1 h after decompression (D and D+GSK2795039 groups) or 1 h after chamber exposure (control group). We also added a brief description of tissue collection and processing.

Changes in the clean manuscript:

- Materials and methods (Tissue collection): [Page 3-4, Lines 91-111]

3. Sample sizes (n):

We now report sample sizes for each assay in the Materials and methods. Vascular reactivity (wire myography) was performed with n = 3 animals per group, and all other measurements were performed with n = 6 animals per group. We also clarify tissue allocation (pulmonary artery segments used for myography were collected separately from tissue used for biochemical/ELISA analyses).

Changes in the clean manuscript:

- Materials and methods (Sample size calculation): [Page 4, Lines 112-129]

*Comment#5. For each of the assays, like ROS, NO - specify the kit or probe used.*

Response:

Thank you for this helpful suggestion. We have revised the Methods to explicitly specify the assay kits/probes (manufacturer and catalog number). ROS was measured using a DCFH-DA fluorescent probe (Nanjing Jiancheng Bioengineering Institute, Nanjing, China; Cat. No. E004-1-1), and NO (NOx) was measured using a NO detection kit (Nanjing Jiancheng Bioengineering Institute, Nanjing, China; Cat. No. A012-1). We also specified the kit information for other biochemical assays (e.g., SOD and MDA) in the same section.

In addition, we provide a complete table listing all kit or probe used in the study (Supporting Information file S3 Reagents, Kits, and Antibodies).

Changes in the clean manuscript:

- Materials and methods (Biochemical assays): [Page 5-6, Lines 162-208]

*Comment#6. For ET-1 assay, it is unclear what was the standard or sample used in the study and the concentration.*

Response:

Thank you for this comment. We have revised the Materials and methods to clarify the ELISA (ET-1, ICAM-1, VCAM-1) procedure, including the kit information, the use of standards and samples, and the calculation/reporting approach. ET-1, ICAM-1, VCAM-1 were measured using a rat ELISA kit. For each well, 100 µL of standard or sample was added to the pre-coated 96-well plate, and ET-1, ICAM-1, VCAM-1 concentrations were determined from the kit standard curve (ET-1 standard concentration range 1.25-80pg/ml, ICAM-1 standard concentration range 78.125-5000 pg/ml, VCAM-1 standard concentration range 156-10000pg/ml). ET-1, ICAM-1, VCAM-1 values were normalized to total protein content and are reported as pg/mg protein. These details have been added under Materials and methods—ELISA assays.

Changes in the clean manuscript:

- Materials and methods (ELISA assays): [Page 13-14, Lines 231-250]

*Comment#7. Overall clarity of the methodology section needs to be improved by specifying details of the sample concentration, the reference number of probes or kits used and the time point after which each of this was done. Also, provide table detailing the primary and secondary antibodies used for the study.*

Response:

Thank you for this constructive comment. We have comprehensively revised the Materials and Methods to improve clarity and reproducibility by explicitly specifying: (i) sample preparation and normalization (including protein quantification and reporting units normalized to protein content), (ii) manufacturer and catalog numbers (Cat. Nos.) for all probes/kits/reagents used, and (iii) the key experimental time points, including post-dive monitoring and the tissue harvest time point (pulmonary arteries collected 1 h after decompression, or 1 h after chamber exposure for controls), as well as the relevant processing/incubation conditions described within each assay subsection (e.g., centrifugation and incubation times/temperatures).

In addition, as requested, we provide a complete table listing all antibodies used in the study. The Supporting Information file S3 Reagents, Kits, and Antibodies summarizes all primary and secondary antibodies, including target, supplier, catalog number, dilution, and is referenced in the revised Materials and Methods.

These revisions are indicated in the Materials and Methods section and Supporting Information (S3 Reagents, Kits, and Antibodies).

Changes in the clean manuscript:

- Materials and methods (Biochemical assays, ELISA assays): [Page 5-6, Lines 162-208; Lines 222-239]

*Comment#8. To address that NADPH oxidase inhibitor reduces endothelial injury, apart from endothelin marker, including additional makers of injury in the context of inflammation or oxidative stress also would add more rigor to the study.*

Response:

Thank you for this valuable suggestion. We agree that additional inflammation- and oxidative stress–related injury markers would strengthen the evidence that NOX2 inhibition reduces endothelial injury. Accordingly, in addition to ET-1, we expanded the revised study to include endothelial activation markers ICAM-1 and VCAM-1 (Fig 4B–C) and oxidative stress–related endpoints including BH4 (Fig 3F) as well as SOD activity and MDA levels (Fig2B-C). These markers were measured from pulmonary artery tissue preparations collected 1 h after decompression (diving groups) or 1 h after chamber exposure (controls), and the corresponding kit/probe information (manufacturer and catalog number), sample preparation, and normalization have been clarified in the Materials and methods. The new data are incorporated into the Results and support a more rigorous assessment of endothelial injury beyond ET-1 alone.

Changes in the clean manuscript:

- Materials and methods (Biochemical assays-SOD/MDA): [Page 5-6, Lines 175-199]
- Materials and methods (ELISA assays): [Page 6, Lines 222-239]
- Results (Fig2B-C; Fig3F; Fig4B-C;): [Page 7-9, Lines 267-337]

*Comment#9. Include limitations of the study.*

Response:

Thank you for this suggestion. We agree that explicitly stating the study limitations improves transparency and interpretation. We have added a dedicated Limitations of the study subsection in the Discussion. Key limitations now stated include: (i) several biochemical endpoints were measured from pulmonary artery whole-vessel/tissue preparations and therefore are not strictly endothelium-specific; (ii) pharmacologic inhibition (GSK2795039) strengthens source-specific inference but does not replace genetic loss-of-function validation (e.g., NOX2 knockout or cell-specific approaches); and (iii) the study focuses on an acute post-decompression time point (1 h), and additional time-course and/or dose–response studies may further refine the mechanistic framework. We also tempered conclusions to align with these limitations. These changes have been added in the Discussion.

Changes in the clean manuscript:

- Limitations of the study: The subsection was added in the Discussion. [Page 10-11, Lines 420-431]
