# Supplementary material for: NOX2 inhibition attenuates oxidative stress and eNOS uncoupling in pulmonary arteries of rats following simulated air diving
Source: PLoS One. 2026 Jul 2;21(7):e0351145. doi: 10.1371/journal.pone.0351145 (PMC13327252; doi:10.1371/journal.pone.0351145)
Supplement: Attachment — Submitted filename: Response_to_Reviewers_auresp_2.docx [file pone.0351145.s006.docx]

Response Letter to PLOS ONE Submission

Paper ID: PONE-D-25-62136

Paper Title: NOX2 inhibition attenuates oxidative stress and eNOS uncoupling in pulmonary arteries of rats following simulated air diving

Dear Academic Editor (Dr. Zhang) and the reviewers:

We thank the editor and reviewers for their constructive comments. We have revised the manuscript to (i) temper mechanistic/causal language, (ii) clarify that several biochemical outcomes are indirect indices and/or vessel-level readouts from whole pulmonary artery homogenates, and (iii) improve readability by clarifying group descriptions, defining key abbreviations at first use, removing repetition, and revising Results headings and figure legends.

Below we respond point-by-point.

We are hopeful that the revisions we have made satisfy the concerns raised by the reviewers and look forward to your positive response.

Sincerely,

Wang Song

Exercise Immunology Center, Wuhan Sports University

Wuhan, China

**Reviewer #1**

*Comment 1: Please temper mechanistic/causal claims (e.g., BH4 and oxidative stress are indirect indices; “eNOS uncoupling” should be described cautiously).*

**Response:**

We thank the Reviewer for this helpful comment. We systematically revised the manuscript to use association-based language and to frame our findings as supportive evidence rather than definitive mechanistic causality. We revised eNOS-related wording to “coupling-related readouts” and/or changes in the dimer/monomer ratio, and added explicit interpretive caveats in the Discussion stating that these endpoints are indirect indices rather than direct proof of uncoupled eNOS activity.

**Changes made (Revised manuscript):**

- **Abstract:** Revised to emphasize association-based interpretation (e.g., “NOX2 is associated with… altered eNOS coupling”; “markers consistent with eNOS coupling”; “markers associated with endothelial activation”; and softened the final concluding statement to avoid “upstream driver” language). (p. 1–2, Abstract)
- **Introduction:** Revised the hypothesis framing to reduce mechanistic overstatement (e.g., “may promote… potentially contributing to… coupling–related readouts”). (p. 4, lines 33–43)
- **Results headings:** Replaced strong causal phrasing with “increases/attenuated/decompression-associated/partially improves/coupling–related readouts”. (p. 14–19, lines 264–367)
- **Discussion:** Added explicit statement that reduced BH4 content, lower eNOS dimer/monomer ratio, and decreased NOx should be interpreted as indirect indices consistent with altered eNOS coupling rather than definitive proof of uncoupled eNOS activity. (p. 22, lines 424–427)

*Comment 2: Oxidative stress assays do not identify specific ROS sources; please clarify* *these limitations and interpret results as indices.*

**Response:**

We agree with this point. We added explicit language in both Methods and Discussion clarifying that the oxidative stress readouts represent indices and do not identify the enzymatic source(s) of ROS.

**Changes made (Revised manuscript):**

- **Methods (ROS assay):** Added: “DCFH-DA fluorescence provides an oxidant-sensitive index and does not specify the enzymatic source(s) of ROS.” (p. 10, lines 170–182)
- **Discussion:** Added clarification that oxidative readouts are indices and do not identify specific enzymatic ROS sources, and expanded interpretation to acknowledge other possible contributors. (p. 21, lines 402–413)

*Comment 3: Please avoid implying endothelial cell–specific conclusions when measurements are derived from whole-vessel homogenates.*

**Response:**

We appreciate this suggestion. We clarified in the Results, Discussion, and Conclusions that several outcomes were quantified in **whole pulmonary artery homogenates** and should be interpreted as vessel-level readouts consistent with endothelial activation/dysfunction, but not as endothelial-cell–specific changes.

**Changes made (Revised manuscript):**

- **Results (NOX2):** Specified “whole-vessel homogenates”. (p. 14, lines 266–268)
- **Results (ET-1/ICAM-1/VCAM-1):** Revised heading and text to “markers associated with endothelial activation in pulmonary artery homogenates” and emphasized “vessel-level changes consistent with endothelial activation.” (p. 18, lines 336–348)
- **Discussion:** Added statement that these proteins were quantified in whole pulmonary artery homogenates and should be interpreted as vessel-level changes consistent with (but not specific to) endothelial activation. (p. 23, lines 446–448)
- **Conclusions:** Added “measured at the vessel level.” (p. 24, lines 465–468)

*Comment 4: Functional evidence should be described as supportive (myography sample size is limited).*

**Response:**

We agree and added a statement in the Discussion noting that the vascular reactivity experiments were performed in a limited number of animals; therefore, the functional findings should be interpreted as supportive rather than definitive.

**Changes made (Revised manuscript):**

- **Discussion:** Added the sample-size/interpretation limitation for myography. (p. 22, lines 435–438)

**Reviewer #2**

*Comment 1: Improve the flow and readability of the manuscript.*

**Response:**

We agree and revised the Methods, Results headings, and figure legends to improve flow and readability, particularly by clarifying grouping/treatments, defining abbreviations, removing redundancy, and aligning headings/legends with measured endpoints and interpretation limits.

**Changes made (Revised manuscript):**

- Readability-related revisions across Methods and Results (p. 6–19), including revised Results headings and figure legends (see details below).

*Comment 2: The experimental grouping description can be improved.*

**Response:**

We agree and rewrote the “Experiment grouping” section to make group differences clearer, explicitly stating chamber exposure conditions and pretreatments (vehicle vs. GSK2795039), including dose/route/timing. We also defined ATA at first use in this section.

**Changes made (Revised manuscript):**

- **Experiment grouping rewritten** (p. 6, lines 72–86)

*Comment 3: Remove repetition (tissue collection timing described in more than one* *section).*

**Response:**

We agree. We removed the redundant tissue-harvest timing sentence from “Simulated diving procedure” and retained tissue collection timing in the “Tissue collection” subsection.

**Changes made (Revised manuscript):**

- **Simulated diving procedure:** Removed the redundant harvest-timing sentence. (p. 6–7, lines 87–94)
- **Tissue collection:** Retained collection timing statement. (p. 7, lines 95–99)

*Comment 4: Define abbreviations at first use (e.g., ATA) and improve consistency.*

**Response:**

We agree. We defined key abbreviations at first mention, including ATA and Emax, and expanded several assay headings to include full terms/definitions for clarity.

**Changes made (Revised manuscript):**

- **ATA:** Defined as “1 atmosphere absolute (ATA)” at first use. (p. 6, lines 73–75)
- **Emax:** Defined as “maximal relaxation (Emax)” at first use. (p. 8, lines 126–127)
- **Assay headings expanded:** e.g., “Reactive oxygen species (ROS) activity assay,” “Malondialdehyde (MDA) assay,” and “NOx (total nitrate/nitrite) assay.” (p. 10–12, lines 170–210)

*Comment 5: Figure legends should clearly define groups C and D.*

**Response:**

We agree and revised figure legends for Figs 1–5 to define group abbreviations (C, D, and D + GSK2795039) directly in each legend.

**Changes made (Revised manuscript):**

- **Fig 1 legend:** (p. 15, lines 272–278)
- **Fig 2 legend:** (p. 16, lines 293–299)
- **Fig 3 legend:** (p. 17, lines 333–335)
- **Fig 4 legend:** (p. 18, lines 354–355)
- **Fig 5 legend:** (p. 19, lines 378–379)
