# Supplementary material for: NOX2 inhibition attenuates oxidative stress and eNOS uncoupling in pulmonary arteries of rats following simulated air diving
Source: PLoS One. 2026 Jul 2;21(7):e0351145. doi: 10.1371/journal.pone.0351145 (PMC13327252; doi:10.1371/journal.pone.0351145)
Supplement: Attachment — Submitted filename: Response_to_Reviewers_auresp_3.docx [file pone.0351145.s007.docx]

**Response Letter to PLOS ONE Submission**

**Manuscript ID:** PONE-D-25-62136R2

**Manuscript title:** NOX2 inhibition attenuates oxidative stress and eNOS uncoupling in pulmonary arteries of rats following simulated air diving

Dear Dr. Zhang and Reviewers,

We sincerely thank the Academic Editor and the reviewers for their careful evaluation of our revised manuscript. We are grateful that both reviewers considered that the major concerns had been addressed. In response to the remaining minor comments from Reviewer #1, we have revised the manuscript and checked the full text, statistical notation, group-name formatting, figure legends, and language. A point-by-point response is provided below. All revisions are reflected in the revised manuscript and the marked-up version.

# Response to Reviewer #1

We thank Reviewer #1 for the careful evaluation of our revised manuscript and for the constructive minor suggestions.

***Comment 1:*** *The letter p should be italicized throughout the statistical analysis section.*

**Response:** Thank you for this suggestion. We have italicized the letter *p* throughout the Statistical analysis section. We also checked the Results section and figure legends to ensure consistent formatting of statistical expressions, including *p*-values.

***Comment 2:*** *Please provide the full name of SOD at its first mention in the manuscript.*

**Response:** Thank you. We have revised the first mention of this abbreviation to “superoxide dismutase (SOD)” and used “SOD” thereafter. This revision is also reflected consistently in the relevant methods subsection and figure legend.

***Comment 3:*** *The manuscript should use a consistent and standard format for group names. For example, “Group A, Group B, Group C, Group D” is preferred over “A/B/C/D group” for clarity and consistency.*

**Response:** We agree with the reviewer. We have carefully checked the manuscript and figure legends and standardized the group designations throughout the text. The groups are now described consistently as Group C, Group D, and Group D + GSK2795039, with definitions provided at first mention as follows: Group C, control group; Group D, decompression-stress group; and Group D + GSK2795039, NOX2 inhibitor-treated decompression-stress group.

***Comment 4:*** *Line 314: The word “and” is used redundantly and should be removed.*

**Response:** Thank you for pointing this out. We have removed the redundant wording in the Conclusions section and revised the sentence to improve clarity and readability.

***Comment 5:*** *The manuscript would benefit from careful language revision by a native English speaker to improve clarity and readability.*

**Response:** Thank you for this helpful suggestion. We have carefully revised the manuscript throughout for language, grammar, punctuation, terminology, clarity, and readability. We also checked the consistency of statistical expressions, group labels, and figure legends.

# Response to Reviewer #2

***Comment:*** *The authors have addressed all major concerns, clearly defined the groups, and significantly improved the clarity of the paper.*

**Response:** We sincerely thank Reviewer #2 for the positive assessment. No additional concerns were raised. We have retained the revisions that addressed the major concerns and conducted an additional final check to ensure consistency, clarity, and readability throughout the manuscript.

We hope that the revised manuscript and the responses above satisfactorily address the remaining comments. We again thank the Academic Editor and reviewers for their time and constructive feedback.

Sincerely,

Wang Song

Exercise Immunology Center, Wuhan Sports University

Wuhan, China
