# Supplementary material for: NOX2 inhibition attenuates oxidative stress and eNOS uncoupling in pulmonary arteries of rats following simulated air diving
Source: PLoS One. 2026 Jul 2;21(7):e0351145. doi: 10.1371/journal.pone.0351145 (PMC13327252; doi:10.1371/journal.pone.0351145)
Supplement: Peer review history

# Peer review: Decision Letter 0

**DOI:** 10.1371/journal.pone.0351145.r001

**Authors:** Peng Zhang

15 Dec 2025

PONE-D-25-62136

NOX2/ROS-mediated eNOS uncoupling leads to endothelial dysfunction in the pulmonary arteries of simulated air diving rats

PLOS One

Dear Dr. Wang,

Thank you for submitting your manuscript to PLOS ONE. After careful consideration, we feel that it has merit but does not fully meet PLOS ONE’s publication criteria as it currently stands. Therefore, we invite you to submit a revised version of the manuscript that addresses the points raised during the review process.

Please submit your revised manuscript by Jan 29 2026 11:59PM. If you will need more time than this to complete your revisions, please reply to this message or contact the journal office at plosone@plos.org. When you're ready to submit your revision, log on to https://www.editorialmanager.com/pone/ and select the 'Submissions Needing Revision' folder to locate your manuscript file.

Please include the following items when submitting your revised manuscript:

- A letter that responds to each point raised by the academic editor and reviewer(s). You should upload this letter as a separate file labeled 'Response to Reviewers'.
- A marked-up copy of your manuscript that highlights changes made to the original version. You should upload this as a separate file labeled 'Revised Manuscript with Track Changes'.
- An unmarked version of your revised paper without tracked changes. You should upload this as a separate file labeled 'Manuscript'.

If you would like to make changes to your financial disclosure, please include your updated statement in your cover letter. Guidelines for resubmitting your figure files are available below the reviewer comments at the end of this letter.

If applicable, we recommend that you deposit your laboratory protocols in protocols.io to enhance the reproducibility of your results. Protocols.io assigns your protocol its own identifier (DOI) so that it can be cited independently in the future. For instructions see: https://journals.plos.org/plosone/s/submission-guidelines#loc-laboratory-protocols. Additionally, PLOS ONE offers an option for publishing peer-reviewed Lab Protocol articles, which describe protocols hosted on protocols.io. Read more information on sharing protocols at https://plos.org/protocols?utm_medium=editorial-email&utm_source=authorletters&utm_campaign=protocols.

We look forward to receiving your revised manuscript.

Kind regards,

Peng Zhang, Ph.D.

Academic Editor

PLOS One

Journal Requirements:

When submitting your revision, we need you to address these additional requirements.

1. Please ensure that your manuscript meets PLOS ONE's style requirements, including those for file naming. The PLOS ONE style templates can be found at

https://journals.plos.org/plosone/s/file?id=wjVg/PLOSOne_formatting_sample_main_body.pdf and

https://journals.plos.org/plosone/s/file?id=ba62/PLOSOne_formatting_sample_title_authors_affiliations.pdf

2. To comply with PLOS One submissions requirements, in your Methods section, please provide additional information regarding the experiments involving animals and ensure you have included details on (1) methods of sacrifice, (2) methods of anesthesia and/or analgesia, and (3) efforts to alleviate suffering.

3. Please update your submission to use the PLOS LaTeX template. The template and more information on our requirements for LaTeX submissions can be found at http://journals.plos.org/plosone/s/latex.

4. Thank you for stating the following in the Acknowledgments Section of your manuscript:

“This work received no external funding from any agency in the public, commercial, or not-for-profit sector”

We note that you have provided funding information that is not currently declared in your Funding Statement. However, funding information should not appear in the Acknowledgments section or other areas of your manuscript. We will only publish funding information present in the Funding Statement section of the online submission form.

Please remove any funding-related text from the manuscript and let us know how you would like to update your Funding Statement. Currently, your Funding Statement reads as follows:

“The author(s) received no specific funding for this work.”

Please include your amended statements within your cover letter; we will change the online submission form on your behalf.

5. We note that your Data Availability Statement is currently as follows: “All relevant data are within the manuscript and its Supporting Information files.”

Please confirm at this time whether or not your submission contains all raw data required to replicate the results of your study. Authors must share the “minimal data set” for their submission. PLOS defines the minimal data set to consist of the data required to replicate all study findings reported in the article, as well as related metadata and methods (https://journals.plos.org/plosone/s/data-availability#loc-minimal-data-set-definition).

For example, authors should submit the following data:

- The values behind the means, standard deviations and other measures reported;

- The values used to build graphs;

- The points extracted from images for analysis.

Authors do not need to submit their entire data set if only a portion of the data was used in the reported study.

If your submission does not contain these data, please either upload them as Supporting Information files or deposit them to a stable, public repository and provide us with the relevant URLs, DOIs, or accession numbers. For a list of recommended repositories, please see https://journals.plos.org/plosone/s/recommended-repositories.

If there are ethical or legal restrictions on sharing a de-identified data set, please explain them in detail (e.g., data contain potentially sensitive information, data are owned by a third-party organization, etc.) and who has imposed them (e.g., an ethics committee). Please also provide contact information for a data access committee, ethics committee, or other institutional body to which data requests may be sent. If data are owned by a third party, please indicate how others may request data access.

6. PLOS ONE now requires that authors provide the original uncropped and unadjusted images underlying all blot or gel results reported in a submission’s figures or Supporting Information files. This policy and the journal’s other requirements for blot/gel reporting and figure preparation are described in detail at https://journals.plos.org/plosone/s/figures#loc-blot-and-gel-reporting-requirements and https://journals.plos.org/plosone/s/figures#loc-preparing-figures-from-image-files. When you submit your revised manuscript, please ensure that your figures adhere fully to these guidelines and provide the original underlying images for all blot or gel data reported in your submission. See the following link for instructions on providing the original image data: https://journals.plos.org/plosone/s/figures#loc-original-images-for-blots-and-gels.

In your cover letter, please note whether your blot/gel image data are in Supporting Information or posted at a public data repository, provide the repository URL if relevant, and provide specific details as to which raw blot/gel images, if any, are not available. Email us at plosone@plos.org if you have any questions.

7. We note that you have included the phrase “data not shown” in your manuscript. Unfortunately, this does not meet our data sharing requirements. PLOS does not permit references to inaccessible data. We require that authors provide all relevant data within the paper, Supporting Information files, or in an acceptable, public repository. Please add a citation to support this phrase or upload the data that corresponds with these findings to a stable repository (such as Figshare or Dryad) and provide and URLs, DOIs, or accession numbers that may be used to access these data. Or, if the data are not a core part of the research being presented in your study, we ask that you remove the phrase that refers to these data.

8. Your ethics statement should only appear in the Methods section of your manuscript. If your ethics statement is written in any section besides the Methods, please delete it from any other section.

9. Please remove your figures from within your manuscript file, leaving only the individual TIFF/EPS image files, uploaded separately. These will be automatically included in the reviewers’ PDF.

10. If the reviewer comments include a recommendation to cite specific previously published works, please review and evaluate these publications to determine whether they are relevant and should be cited. There is no requirement to cite these works unless the editor has indicated otherwise.

[Note: HTML markup is below. Please do not edit.]

Reviewers' comments:

Reviewer's Responses to Questions

**Comments to the Author**

1. Is the manuscript technically sound, and do the data support the conclusions?

The manuscript must describe a technically sound piece of scientific research with data that supports the conclusions. Experiments must have been conducted rigorously, with appropriate controls, replication, and sample sizes. The conclusions must be drawn appropriately based on the data presented.

Reviewer #1: Partly

Reviewer #2: Yes

**********

2. Has the statistical analysis been performed appropriately and rigorously?

Reviewer #1: Yes

Reviewer #2: Yes

**********

3. Have the authors made all data underlying the findings in their manuscript fully available?

The PLOS Data policy requires authors to make all data underlying the findings described in their manuscript fully available without restriction, with rare exception (please refer to the Data Availability Statement in the manuscript PDF file). The data should be provided as part of the manuscript or its supporting information, or deposited to a public repository. For example, in addition to summary statistics, the data points behind means, medians and variance measures should be available. If there are restrictions on publicly sharing data—e.g. participant privacy or use of data from a third party—those must be specified.

Reviewer #1: Yes

Reviewer #2: Yes

**********

4. Is the manuscript presented in an intelligible fashion and written in standard English?

PLOS ONE does not copyedit accepted manuscripts, so the language in submitted articles must be clear, correct, and unambiguous. Any typographical or grammatical errors should be corrected at revision, so please note any specific errors here.

Reviewer #1: No

Reviewer #2: Yes

**********

5. Review Comments to the Author

Please use the space provided to explain your answers to the questions above. You may also include additional comments for the author, including concerns about dual publication, research ethics, or publication ethics. (Please upload your review as an attachment if it exceeds 20,000 characters)

Reviewer #1: The manuscript investigates NOX2-mediated oxidative stress and eNOS uncoupling in a rat diving model. While the research question is potentially interesting, the study in its current form is not suitable for publication and would require extensive additional experimentation and substantial rewriting to meet the scientific and editorial standards of the journal.

1. Novelty is limited and not convincingly demonstrated.

Although the authors attempt to connect NOX2-derived ROS with eNOS uncoupling during simulated diving, both pathways are already well described, and the manuscript does not provide compelling evidence that the findings represent a novel mechanism specific to decompression-related vascular injury.

2. Mechanistic evidence is insufficient.

The study presents only associative data linking NOX2, ROS, and eNOS uncoupling. No deeper causal validation is provided (e.g., NOX2 knockout models, genetic or pharmacologic rescue beyond apocynin). The reliance on apocynin—an agent with broad antioxidant effects and limited NOX2 specificity—weakens the mechanistic conclusions.

Additionally, the manuscript lacks functional vascular assays (e.g., endothelium-dependent vasodilation tests), which are essential to substantiate the claim of “endothelial dysfunction.”

3. Concerns regarding assay methodology.

The ROS, NO, and ET-1 assays are performed on whole vessel tissue rather than isolated endothelium or primary endothelial cells. If this approach represents an accepted standard for the diving-related vascular field, appropriate references must be provided. Otherwise, the measurements may not accurately reflect endothelial-specific changes.

4. The Discussion requires major restructuring.

The writing contains redundancies, abrupt shifts between topics, and several unclear explanations. The lack of logical flow significantly diminishes the readability and interpretability of the findings.

Given these scientific and editorial issues, the manuscript requires major revision and additional mechanistic data before it can be suitable for publication.

Reviewer #2: Qing Bo and group in the manuscript titled ‘NOX2/ROS-mediated eNOS uncoupling leads to endothelial dysfunction in the pulmonary arteries of simulated air diving rats’ have used a rat model to demonstrate how the eNOS uncoupling caused by increased NOX-2 mediated ROS production progresses to endothelial dysfunction. Three groups have been used in the study: control group, test group, and apocynin treated test group. The test group mice were placed in pressurized chamber followed by gradual decompression to mimic the effects in diving environment. Following this, various analysis on the pulmonary artery collected have been used to determine the changes between groups.

The following needs to be addressed:

1. In the simulated diving procedure, mention the normal pressure range of the pressurized chamber in which control animals were housed.

2. Specify the rat strain used for the study.

3. Intraperitoneal injection of apocynin: was it just a single dose before subjecting to diving procedure.

4. Elaborate on the behavioral indicators observed after the simulated diving procedure other than analysis done using pulmonary artery tissue sample. How long after the procedure was this done and also include details on pulmonary artery tissue collection and the time point. How many tissues have been used for each of the analysis performed?

5. For each of the assays, like ROS, NO - specify the kit or probe used.

6. For ET-1 assay, it is unclear what was the standard or sample used in the study and the concentration.

7. Overall clarity of the methodology section needs to be improved by specifying details of the sample concentration, the reference number of probes or kits used and the time point after which each of this was done. Also, provide table detailing the primary and secondary antibodies used for the study

8. To address that NADPH oxidase inhibitor reduces endothelial injury, apart from endothelin marker, including additional makers of injury in the context of inflammation or oxidative stress also would add more rigor to the study.

9. Include limitations of the study.

**********

6. PLOS authors have the option to publish the peer review history of their article (what does this mean?). If published, this will include your full peer review and any attached files.

If you choose “no”, your identity will remain anonymous but your review may still be made public.

**Do you want your identity to be public for this peer review?**  For information about this choice, including consent withdrawal, please see our Privacy Policy.

Reviewer #1: No

Reviewer #2: No

**********

[NOTE: If reviewer comments were submitted as an attachment file, they will be attached to this email and accessible via the submission site. Please log into your account, locate the manuscript record, and check for the action link "View Attachments". If this link does not appear, there are no attachment files.]

To ensure your figures meet our technical requirements, please review our figure guidelines: https://journals.plos.org/plosone/s/figures

You may also use PLOS’s free figure tool, NAAS, to help you prepare publication quality figures: https://journals.plos.org/plosone/s/figures#loc-tools-for-figure-preparation.

NAAS will assess whether your figures meet our technical requirements by comparing each figure against our figure specifications.

# Author comment: Author response to Decision Letter 1

**DOI:** 10.1371/journal.pone.0351145.r002

28 Jan 2026

Dear Academic Editor (Dr. Zhang) and the reviewers:

Thank you for the opportunity to revise our manuscript. We sincerely thank the editor and all reviewers for their valuable feedback that we have used to improve the quality of our manuscript.

During the major revision, Reviewer 1 raised an important methodological concern regarding the limited specificity of the inhibitor used in the original submission. To directly address this key issue and to improve the reliability and interpretability of our conclusions, we replaced the inhibitor in the third group with GSK2795039 (the reagent information and dosing regimen have been updated in the Materials and methods section).

Given that the key intervention factor was changed, to avoid potential confounding introduced by different animal batches and/or experimental time periods and to ensure comparability among the three groups, we conducted a complete repeat of the experiments using an independent new animal cohort for the control, decompression, and inhibitor-treated groups, and remeasured all original endpoints. In addition, following the reviewers’ suggestions, we added/supplemented measurements of ICAM-1, VCAM-1, BH4,SOD, and MDA, as well as functional assays using pulmonary artery rings, to strengthen the mechanistic and functional evidence.

All data presented in the revised manuscript are derived from the repeated experiments in this new cohort; the corresponding figures, statistical analyses, and supplementary materials have been updated accordingly. All additional experiments were performed within the scope of the original ethical approval.

We submitted 3 files, including a revised manuscript, a track changes file (to highlight differences between the revised and the original manuscript), and this file, i.e., the response letter. In the following, we respond to each of your concerns and recommendations point-by-point. All changes are shown in the tracked-changes file, and corresponding locations are provided for the clean manuscript.

We are hopeful that the revisions we have made satisfy the concerns raised by the reviewers and look forward to your positive response.

Sincerely,

Wang Song

Exercise Immunology Center, Wuhan Sports University

Wuhan, China

Response to the Editor #

Journal Requirement 1 (Style requirements and file naming)

Comment#1. Please ensure that your manuscript meets PLOS ONE's style requirements, including those for file naming.

Response:

We have revised the manuscript to comply with PLOS ONE style and formatting requirements (including section structure, figure legends, and reference formatting) and ensured that all files follow the requested naming conventions. In the resubmission package, we provide separate files for (i) the point-by-point response, (ii) the tracked-changes manuscript, and (iii) the clean revised manuscript. Figures are provided as separate high-resolution TIFF files, and Supporting Information files are provided separately as required.

Files submitted (named as requested):

• Response to Reviewers: Response to Reviewers

• Revised Manuscript with Track Changes: Revised Manuscript with Track Changes(docx)

• Clean Manuscript: Manuscript (PDF)

• Cover letter: Cover letter (Revised) (.docx)

• Figure files: Fig1, Fig2, Fig3, Fig4, Fig5 (TIFF)

• Supporting Information: S1 Data(xlsx); S2 Raw image (PDF); S3 Reagents, Kits, and Antibodies(xlsx)

Journal Requirement 2 (Animal experiments details in Methods)

Comment#2. To comply with PLOS ONE submissions requirements, in your Materials and methods section, please provide additional information regarding the experiments involving animals and ensure you have included details on (1) methods of sacrifice, (2) methods of anesthesia and/or analgesia, and (3) efforts to alleviate suffering.

Response：

Thank you for your comment. We have revised the Materials and methods section to provide the requested animal welfare and procedural details.

(1) Methods of sacrifice/euthanasia:

Rats were euthanized while under deep anesthesia by exsanguination via cardiac puncture followed by thoracotomy. Death was confirmed by cessation of heartbeat and respiration.

(2) Anesthesia/analgesia:

Before tissue collection, rats were deeply anesthetized with sodium pentobarbital (50 mg/kg, i.p., with supplemental doses as needed). Adequate depth of anesthesia was confirmed by the absence of pedal withdrawal and corneal reflexes. We also specified the predefined rescue analgesia plan: buprenorphine (0.05 mg/kg, subcutaneously) would have been administered if persistent pain/distress had occurred; however, no animal reached a humane endpoint and buprenorphine was not administered in this study.

(3) Efforts to alleviate suffering:

Animals were acclimatized for at least one week prior to experimentation and were monitored daily for general appearance, body weight, grooming behavior, and respiratory pattern. During and after the simulated diving procedure, rats were closely observed for signs of discomfort or neurological symptoms (e.g., labored breathing, tremor, paralysis, or prolonged recumbency). Predefined humane endpoints and immediate euthanasia under deep anesthesia were in place if persistent distress had been observed.

Changes in the clean manuscript:

• Materials and methods (Ethics statement): [Page 4-5, Lines 42–66]

Journal Requirement 3 (Use the PLOS LaTeX template)

Comment#3. Please update your submission to use the PLOS LaTeX template.

Response:

Thank you for the instruction. We have converted the revised manuscript to the PLOS LaTeX template and are providing the compiled PDF, as part of the resubmission. Figures are uploaded separately as high-resolution TIFF files in accordance with PLOS ONE requirements.

Files provided:

• Compiled PDF: Manuscript (LaTeX PDF)

Journal Requirement 4 (Funding information placement and Funding Statement update)

Comment#4.Remove any funding-related text from the manuscript and indicate how you would like to update the Funding Statement; include amended statements in the cover letter.

Response:

We have removed all funding-related text from the manuscript (including the “Funding” section) and confirm that funding information should appear only in the online Funding Statement.

Changes in the Requested Funding Statement:

• “The authors received no specific funding for this work.”

Changes in the Cover letter:

• We have included the amended Funding Statement exactly as written above.

Journal Requirement 5 (Minimal data set / Data Availability confirmation)

Comment#5. We note that your Data Availability Statement is currently as follows: “All relevant data are within the manuscript and its Supporting Information files.”

Please confirm at this time whether or not your submission contains all raw data required to replicate the results of your study. Authors must share the “minimal data set” for their submission.

Response：

We confirm that the minimal data set underlying the reported findings is fully available. All underlying/raw values required to reproduce the analyses and figures are provided as Supporting Information.

Specifically, S1 Data (xlsx) contains the underlying values for Fig 1–Fig 5, including the raw/underlying measurements used for summary statistics, the data used to generate graphs, and the quantitative values underlying any image-based analyses. These data enable replication of the reported statistical analyses and results.

Updated Data Availability Statement:

All relevant data are within the manuscript and its Supporting Information files. The underlying data for Fig 1–Fig 5 are provided in S1 Data (xlsx), blot images are provided in S2 Raw image (PDF).

Changes in the clean manuscript:

• Supporting Information: S1 Data and S2 Raw image.

Journal Requirement 6 (Original uncropped and unadjusted blot/gel images)

Comment#6. PLOS ONE now requires that authors provide the original uncropped and unadjusted images underlying all blot or gel results reported in a submission’s figures or Supporting Information files.

In your cover letter, please note whether your blot/gel image data are in Supporting Information.

Response：

We have provided the original uncropped and unadjusted images underlying all blot results (Fig 1 and Fig 3) reported in the manuscript. The raw images corresponding to the blots in Fig1 and Fig3 are included in the Supporting Information file S2 Raw image.

Changes in the Cover letter:

• We noted that the original uncropped and unadjusted blot images are provided in Supporting Information (S2 Raw image).

Journal Requirement 7 (“data not shown”)

Comment#7. “data not shown” is not permitted; provide accessible data, add a citation, or remove the phrase if not core.

Response：

Thank you for noting this. We have removed the phrase “data not shown” from the manuscript. All data underlying the findings reported in this study are provided in the submission as Supporting Information (raw data files; see S1 Data), and no references to inaccessible data remain.

Journal Requirement 8 (Ethics statement only in Methods)

Comment#8. Your ethics statement should only appear in the Methods section of your manuscript. If your ethics statement is written in any section besides the Methods, please delete it from any other section.

Response：

We confirm that the ethics statement now appears only in the Materials and methods section. Any duplicate ethics statements that previously appeared outside the Materials and methods section have been removed to comply with PLOS ONE requirements.

Journal Requirement 9 (Remove figures from within manuscript file)

Comment#9. Please remove your figures from within your manuscript file, leaving only the individual TIFF/EPS image files, uploaded separately. These will be automatically included in the reviewers’ PDF.

Response：

We have removed all embedded figures from the manuscript file. All figures are provided as separate figure files (Fig1–Fig5) uploaded individually, while the manuscript contains only the figure callouts and figure legends.

Journal Requirement 10 (Reviewer-suggested citations)

Comment#10. If the reviewer comments include a recommendation to cite specific previously published works, please review and evaluate these publications to determine whether they are relevant and should be cited. There is no requirement to cite these works unless the editor has indicated otherwise.

Response:

In response to the reviewer’s concern about using whole-vessel tissue measurements (ROS, NO, and ET-1) rather than isolated endothelium or primary endothelial cells, we recognized that additional supporting references would strengthen the methodological justification. We therefore added relevant citations in the Methods to support the use of whole-vessel readouts in related decompression/vascular injury contexts, and we clarified the rationale as well as the limitation that these measurements are not endothelium-specific. We also tempered interpretation accordingly in the Results/Discussion.

Changes in the clean manuscript:

• Materials and methods (Tissue collection): [Page 3, Lines 91–111]

Response to Reviewer #1

Comment#1. Novelty is limited and not convincingly demonstrated.

Although the authors attempt to connect NOX2-derived ROS with eNOS uncoupling during simulated diving, both pathways are already well described, and the manuscript does not provide compelling evidence that the findings represent a novel mechanism specific to decompression-related vascular injury.

Response:

We thank the reviewer for the comment regarding novelty. We agree that NOX2-derived ROS signaling and BH4-dependent eNOS uncoupling are well established in chronic vascular disease. Our study does not claim novelty of these pathways per se; rather, it tests in an acute simulated air-diving/decompression model whether a defined enzymatic ROS source (NOX2) contributes to BH4 loss/eNOS uncoupling and pulmonary vascular dysfunction.

To address this concern, we revised the Introduction and Discussion to more clearly define the knowledge gap in the decompression setting and to avoid overstating novelty. Specifically, we now emphasize that prior diving/decompression studies often relied on systemic oxidative indices or nonspecific antioxidant interventions, whereas source-specific evidence linking NOX2 activation to BH4 depletion, eNOS uncoupling, reduced NO bioavailability, and functional impairment in pulmonary arteries has been limited. We also tempered language throughout the Abstract/Conclusion (e.g., replacing “central role/first evidence” with “evidence consistent with a NOX2-dependent contribution”).

Experimentally, the revised study integrates biochemical endpoints (NOX2, ROS/oxidative indices, BH4, NO metabolites, and eNOS coupling status) with wire-myography assessment of endothelium-dependent relaxation and includes pharmacologic NOX2 inhibition (GSK2795039). The concordant improvements in molecular and functional readouts with NOX2 inhibition support a NOX2-dependent contribution to post-decompression endothelial dysfunction in this model.

Changes in the clean manuscript:

• Introduction: revised to clarify the decompression-setting knowledge gap and avoid novelty overstatements. [Page 1-2, Lines 1-40]

• Discussion: revised opening and framing to align with the decompression model and temper claims. [Page 9-11, Lines 361-431]

• Abstract/Conclusion: revised to use cautious language consistent with the evidence. [Page 1; Page 11, Lines 432–442]

Comment#2. Mechanistic evidence is insufficient.

The study presents only associative data linking NOX2, ROS, and eNOS uncoupling. No deeper causal validation is provided (e.g., NOX2 knockout models, genetic or pharmacologic rescue beyond apocynin). The reliance on apocynin—an agent with broad antioxidant effects and limited NOX2 specificity—weakens the mechanistic conclusions.

Additionally, the manuscript lacks functional vascular assays (e.g., endothelium-dependent vasodilation tests), which are essential to substantiate the claim of “endothelial dysfunction.”

Response:

We thank the reviewer for this important critique and agree that the original submission was limited by (i) reliance on apocynin, which has limited NOX2 specificity, and (ii) the lack of functional vascular assays to support “endothelial dysfunction.”

To address these concerns, we substantially strengthened the revision as follows:

1. Improved NOX2 specificity: We replaced apocynin with the NOX2 inhibitor GSK2795039 administered prior to pressurization (supported by prior studies for NOX2 inhibition; Hirano et al., 2015; Oliveira et al., 2022) and updated all Methods/Results accordingly. NOX2 inhibition attenuated decompression-induced oxidative stress and improved NO-related readouts.

Changes in the clean manuscript:

Materials and methods (Experimental grouping): [Page 3, Lines 67-81]

Results (Fig1-Fig5): [Page 7-9, Lines 252-360]

Discussion: [Page 19, Lines 361-431]

2. Mechanistically informative intermediate (BH4): We added direct quantification of tetrahydrobiopterin (BH4) (Fig 3F), a key cofactor whose depletion is consistent with eNOS uncoupling, providing a mechanistically relevant link between oxidative stress and eNOS coupling/NO signaling.

Changes in the clean manuscript:

• Introduction: [Page 2, Lines 16-26]

• Materials and methods (ELISA assays): [Page 6, Lines222-239]

• Results (Fig 3F): [Page 9, Lines 297-304]

• Discussion: [Page 10, Lines 391-401]

3. Functional vascular validation: We added wire-myography vascular reactivity assays, including endothelium-dependent vasodilation (ACh) and endothelium-independent vasodilation (SNP) (Fig 5A–D), providing direct functional evidence of endothelial dysfunction and helping distinguish endothelial impairment from altered smooth muscle responsiveness.

Changes in the clean manuscript:

• Materials and methods (Assessment of vascular endothelial function): [Page 6, Lines 209-221]

• Results (Fig 5A–D): [Page 9, Lines 338-360]

• Discussion: [Page 10, Lines 402-410]

4. Expanded endothelial phenotype markers: We added ICAM-1 and VCAM-1 as established markers of endothelial activation/inflammation (Fig 4B–C), supporting the biological relevance of the observed endothelial injury phenotype.

Changes in the clean manuscript:

• Mate

Attachment Submitted filename: Response to Reviewers.docx

# Peer review: Decision Letter 1

**DOI:** 10.1371/journal.pone.0351145.r003

**Authors:** Peng Zhang

2 Mar 2026

PONE-D-25-62136R1

NOX2 inhibition attenuates oxidative stress and eNOS uncoupling in pulmonary arteries of rats following simulated air diving

PLOS One

Dear Dr. Wang,

Thank you for submitting your manuscript to PLOS ONE. After careful consideration, we feel that it has merit but does not fully meet PLOS ONE’s publication criteria as it currently stands. Therefore, we invite you to submit a revised version of the manuscript that addresses the points raised during the review process.

Please submit your revised manuscript by Apr 16 2026 11:59PM. If you will need more time than this to complete your revisions, please reply to this message or contact the journal office at plosone@plos.org. When you're ready to submit your revision, log on to https://www.editorialmanager.com/pone/ and select the 'Submissions Needing Revision' folder to locate your manuscript file.

Please include the following items when submitting your revised manuscript:

- A letter that responds to each point raised by the academic editor and reviewer(s). You should upload this letter as a separate file labeled 'Response to Reviewers'.
- A marked-up copy of your manuscript that highlights changes made to the original version. You should upload this as a separate file labeled 'Revised Manuscript with Track Changes'.
- An unmarked version of your revised paper without tracked changes. You should upload this as a separate file labeled 'Manuscript'.

-->If you would like to make changes to your financial disclosure, please include your updated statement in your cover letter. Guidelines for resubmitting your figure files are available below the reviewer comments at the end of this letter.

If applicable, we recommend that you deposit your laboratory protocols in protocols.io to enhance the reproducibility of your results. Protocols.io assigns your protocol its own identifier (DOI) so that it can be cited independently in the future. For instructions see: https://journals.plos.org/plosone/s/submission-guidelines#loc-laboratory-protocols. Additionally, PLOS ONE offers an option for publishing peer-reviewed Lab Protocol articles, which describe protocols hosted on protocols.io. Read more information on sharing protocols at https://plos.org/protocols?utm_medium=editorial-email&utm_source=authorletters&utm_campaign=protocols.

We look forward to receiving your revised manuscript.

Kind regards,

Peng Zhang, Ph.D.

Academic Editor

PLOS One

Journal Requirements:

1. If the reviewer comments include a recommendation to cite specific previously published works, please review and evaluate these publications to determine whether they are relevant and should be cited. There is no requirement to cite these works unless the editor has indicated otherwise.

2. Please review your reference list to ensure that it is complete and correct. If you have cited papers that have been retracted, please include the rationale for doing so in the manuscript text, or remove these references and replace them with relevant current references. Any changes to the reference list should be mentioned in the rebuttal letter that accompanies your revised manuscript. If you need to cite a retracted article, indicate the article’s retracted status in the References list and also include a citation and full reference for the retraction notice.

[Note: HTML markup is below. Please do not edit.]

Reviewer's Responses to Questions

**Comments to the Author**

1. If the authors have adequately addressed your comments raised in a previous round of review and you feel that this manuscript is now acceptable for publication, you may indicate that here to bypass the “Comments to the Author” section, enter your conflict of interest statement in the “Confidential to Editor” section, and submit your "Accept" recommendation.

Reviewer #1: All comments have been addressed

Reviewer #2: All comments have been addressed

**********

2. Is the manuscript technically sound, and do the data support the conclusions?

The manuscript must describe a technically sound piece of scientific research with data that supports the conclusions. Experiments must have been conducted rigorously, with appropriate controls, replication, and sample sizes. The conclusions must be drawn appropriately based on the data presented.

Reviewer #1: Yes

Reviewer #2: Yes

**********

3. Has the statistical analysis been performed appropriately and rigorously?

Reviewer #1: Yes

Reviewer #2: Yes

**********

4. Have the authors made all data underlying the findings in their manuscript fully available?

The PLOS Data policy requires authors to make all data underlying the findings described in their manuscript fully available without restriction, with rare exception (please refer to the Data Availability Statement in the manuscript PDF file). The data should be provided as part of the manuscript or its supporting information, or deposited to a public repository. For example, in addition to summary statistics, the data points behind means, medians and variance measures should be available. If there are restrictions on publicly sharing data—e.g. participant privacy or use of data from a third party—those must be specified.

Reviewer #1: Yes

Reviewer #2: Yes

**********

5. Is the manuscript presented in an intelligible fashion and written in standard English?

PLOS ONE does not copyedit accepted manuscripts, so the language in submitted articles must be clear, correct, and unambiguous. Any typographical or grammatical errors should be corrected at revision, so please note any specific errors here.

Reviewer #1: Yes

Reviewer #2: Yes

**********

6. Review Comments to the Author

Please use the space provided to explain your answers to the questions above. You may also include additional comments for the author, including concerns about dual publication, research ethics, or publication ethics. (Please upload your review as an attachment if it exceeds 20,000 characters)

Reviewer #1: The authors have undertaken a substantial revision that addresses the principal scientific concerns raised during the initial review. The revised manuscript demonstrates clear improvement in experimental rigor and conceptual framing. In particular, the replacement of apocynin with a more selective NOX2 inhibitor, the addition of mechanistic intermediates, incorporation of vascular functional assays, and expanded discussion of methodological limitations represent genuine experimental and interpretive advances. These revisions significantly improve the internal coherence of the study and strengthen the link between molecular observations and functional outcomes.

Although the study continues to rely primarily on pharmacologic and associative evidence, the revised dataset now supports a coherent and defensible framework connecting oxidative signaling with vascular dysfunction within the constraints of the model. Importantly, the authors have moderated claims regarding novelty and mechanistic causality, which improves the balance between evidence and interpretation.

Several issues remain that should be addressed to ensure scientific precision. Mechanistic language should consistently reflect that BH4 measurements and oxidative indices are indirect indicators rather than direct proof of eNOS uncoupling. The vascular reactivity experiments are based on a limited sample size and would benefit from a brief clarification that these findings serve as supportive functional evidence. In addition, interpretations derived from whole-vessel biochemical assays should avoid implying endothelial specificity beyond what the data directly support.

These remaining concerns relate to clarity and interpretive rigor rather than core experimental validity. Given the substantial improvements and the now-aligned scope of the conclusions, the manuscript is suitable for publication pending minor revisions addressing the points above.

Reviewer #2: • The authors have addressed all major concerns and have significantly revised and expanded the experimental section. However, the flow and readability of the manuscript could be improved.

• The way in which the experimental grouping has been described can be improved.

• There is some repetition in the text, for instance, the collection of pulmonary artery tissue is mentioned in both Diving section and tissue collection section.

• For clarity, units should be spelled out at first use wherever used(Example: ATA), with the abbreviation provided in parentheses before using in subsequent references.

• The figure legend should clearly define groups C and D as presented in the figure.

**********

7. PLOS authors have the option to publish the peer review history of their article (what does this mean?). If published, this will include your full peer review and any attached files.

If you choose “no”, your identity will remain anonymous but your review may still be made public.

**Do you want your identity to be public for this peer review?**  For information about this choice, including consent withdrawal, please see our Privacy Policy.

Reviewer #1: No

Reviewer #2: No

**********

[NOTE: If reviewer comments were submitted as an attachment file, they will be attached to this email and accessible via the submission site. Please log into your account, locate the manuscript record, and check for the action link "View Attachments". If this link does not appear, there are no attachment files.]

To ensure your figures meet our technical requirements, please review our figure guidelines: https://journals.plos.org/plosone/s/figures

You may also use PLOS’s free figure tool, NAAS, to help you prepare publication quality figures: https://journals.plos.org/plosone/s/figures#loc-tools-for-figure-preparation.

NAAS will assess whether your figures meet our technical requirements by comparing each figure against our figure specifications.

# Author comment: Author response to Decision Letter 2

**DOI:** 10.1371/journal.pone.0351145.r004

31 Mar 2026

Paper ID: PONE-D-25-62136

Paper Title: NOX2 inhibition attenuates oxidative stress and eNOS uncoupling in pulmonary arteries of rats following simulated air diving

Dear Academic Editor (Dr. Zhang) and the reviewers:

We thank the editor and reviewers for their constructive comments. We have revised the manuscript to (i) temper mechanistic/causal language, (ii) clarify that several biochemical outcomes are indirect indices and/or vessel-level readouts from whole pulmonary artery homogenates, and (iii) improve readability by clarifying group descriptions, defining key abbreviations at first use, removing repetition, and revising Results headings and figure legends.

Below we respond point-by-point.

We are hopeful that the revisions we have made satisfy the concerns raised by the reviewers and look forward to your positive response.

Sincerely,

Wang Song

Exercise Immunology Center, Wuhan Sports University

Wuhan, China

Reviewer #1

Comment 1: Please temper mechanistic/causal claims (e.g., BH4 and oxidative stress are indirect indices; “eNOS uncoupling” should be described cautiously).

Response:

We thank the Reviewer for this helpful comment. We systematically revised the manuscript to use association-based language and to frame our findings as supportive evidence rather than definitive mechanistic causality. We revised eNOS-related wording to “coupling-related readouts” and/or changes in the dimer/monomer ratio, and added explicit interpretive caveats in the Discussion stating that these endpoints are indirect indices rather than direct proof of uncoupled eNOS activity.

Changes made (Revised manuscript):

• Abstract: Revised to emphasize association-based interpretation (e.g., “NOX2 is associated with… altered eNOS coupling”; “markers consistent with eNOS coupling”; “markers associated with endothelial activation”; and softened the final concluding statement to avoid “upstream driver” language). (p. 1–2, Abstract)

• Introduction: Revised the hypothesis framing to reduce mechanistic overstatement (e.g., “may promote… potentially contributing to… coupling–related readouts”). (p. 4, lines 33–43)

• Results headings: Replaced strong causal phrasing with “increases/attenuated/decompression-associated/partially improves/coupling–related readouts”. (p. 14–19, lines 264–367)

• Discussion: Added explicit statement that reduced BH4 content, lower eNOS dimer/monomer ratio, and decreased NOx should be interpreted as indirect indices consistent with altered eNOS coupling rather than definitive proof of uncoupled eNOS activity. (p. 22, lines 424–427)

Comment 2: Oxidative stress assays do not identify specific ROS sources; please clarify these limitations and interpret results as indices.

Response:

We agree with this point. We added explicit language in both Methods and Discussion clarifying that the oxidative stress readouts represent indices and do not identify the enzymatic source(s) of ROS.

Changes made (Revised manuscript):

• Methods (ROS assay): Added: “DCFH-DA fluorescence provides an oxidant-sensitive index and does not specify the enzymatic source(s) of ROS.” (p. 10, lines 170–182)

• Discussion: Added clarification that oxidative readouts are indices and do not identify specific enzymatic ROS sources, and expanded interpretation to acknowledge other possible contributors. (p. 21, lines 402–413)

Comment 3: Please avoid implying endothelial cell–specific conclusions when measurements are derived from whole-vessel homogenates.

Response:

We appreciate this suggestion. We clarified in the Results, Discussion, and Conclusions that several outcomes were quantified in whole pulmonary artery homogenates and should be interpreted as vessel-level readouts consistent with endothelial activation/dysfunction, but not as endothelial-cell–specific changes.

Changes made (Revised manuscript):

• Results (NOX2): Specified “whole-vessel homogenates”. (p. 14, lines 266–268)

• Results (ET-1/ICAM-1/VCAM-1): Revised heading and text to “markers associated with endothelial activation in pulmonary artery homogenates” and emphasized “vessel-level changes consistent with endothelial activation.” (p. 18, lines 336–348)

• Discussion: Added statement that these proteins were quantified in whole pulmonary artery homogenates and should be interpreted as vessel-level changes consistent with (but not specific to) endothelial activation. (p. 23, lines 446–448)

• Conclusions: Added “measured at the vessel level.” (p. 24, lines 465–468)

Comment 4: Functional evidence should be described as supportive (myography sample size is limited).

Response:

We agree and added a statement in the Discussion noting that the vascular reactivity experiments were performed in a limited number of animals; therefore, the functional findings should be interpreted as supportive rather than definitive.

Changes made (Revised manuscript):

• Discussion: Added the sample-size/interpretation limitation for myography. (p. 22, lines 435–438)

Reviewer #2

Comment 1: Improve the flow and readability of the manuscript.

Response:

We agree and revised the Methods, Results headings, and figure legends to improve flow and readability, particularly by clarifying grouping/treatments, defining abbreviations, removing redundancy, and aligning headings/legends with measured endpoints and interpretation limits.

Changes made (Revised manuscript):

• Readability-related revisions across Methods and Results (p. 6–19), including revised Results headings and figure legends (see details below).

Comment 2: The experimental grouping description can be improved.

Response:

We agree and rewrote the “Experiment grouping” section to make group differences clearer, explicitly stating chamber exposure conditions and pretreatments (vehicle vs. GSK2795039), including dose/route/timing. We also defined ATA at first use in this section.

Changes made (Revised manuscript):

• Experiment grouping rewritten (p. 6, lines 72–86)

Comment 3: Remove repetition (tissue collection timing described in more than one section).

Response:

We agree. We removed the redundant tissue-harvest timing sentence from “Simulated diving procedure” and retained tissue collection timing in the “Tissue collection” subsection.

Changes made (Revised manuscript):

• Simulated diving procedure: Removed the redundant harvest-timing sentence. (p. 6–7, lines 87–94)

• Tissue collection: Retained collection timing statement. (p. 7, lines 95–99)

Comment 4: Define abbreviations at first use (e.g., ATA) and improve consistency.

Response:

We agree. We defined key abbreviations at first mention, including ATA and Emax, and expanded several assay headings to include full terms/definitions for clarity.

Changes made (Revised manuscript):

• ATA: Defined as “1 atmosphere absolute (ATA)” at first use. (p. 6, lines 73–75)

• Emax: Defined as “maximal relaxation (Emax)” at first use. (p. 8, lines 126–127)

• Assay headings expanded: e.g., “Reactive oxygen species (ROS) activity assay,” “Malondialdehyde (MDA) assay,” and “NOx (total nitrate/nitrite) assay.” (p. 10–12, lines 170–210)

Comment 5: Figure legends should clearly define groups C and D.

Response:

We agree and revised figure legends for Figs 1–5 to define group abbreviations (C, D, and D + GSK2795039) directly in each legend.

Changes made (Revised manuscript):

• Fig 1 legend: (p. 15, lines 272–278)

• Fig 2 legend: (p. 16, lines 293–299)

• Fig 3 legend: (p. 17, lines 333–335)

• Fig 4 legend: (p. 18, lines 354–355)

• Fig 5 legend: (p. 19, lines 378–379)

Attachment Submitted filename: Response_to_Reviewers_auresp_2.docx

# Peer review: Decision Letter 2

**DOI:** 10.1371/journal.pone.0351145.r005

**Authors:** Peng Zhang

24 Apr 2026

PONE-D-25-62136R2

NOX2 inhibition attenuates oxidative stress and eNOS uncoupling in pulmonary arteries of rats following simulated air diving

PLOS One

Dear Dr. Wang,

Thank you for submitting your manuscript to PLOS ONE. After careful consideration, we feel that it has merit but does not fully meet PLOS ONE’s publication criteria as it currently stands. Therefore, we invite you to submit a revised version of the manuscript that addresses the points raised during the review process.

Please submit your revised manuscript by Jun 08 2026 11:59PM. If you will need more time than this to complete your revisions, please reply to this message or contact the journal office at plosone@plos.org. When you're ready to submit your revision, log on to https://www.editorialmanager.com/pone/ and select the 'Submissions Needing Revision' folder to locate your manuscript file.

Please include the following items when submitting your revised manuscript:

- A letter that responds to each point raised by the academic editor and reviewer(s). You should upload this letter as a separate file labeled 'Response to Reviewers'.
- A marked-up copy of your manuscript that highlights changes made to the original version. You should upload this as a separate file labeled 'Revised Manuscript with Track Changes'.
- An unmarked version of your revised paper without tracked changes. You should upload this as a separate file labeled 'Manuscript'.

If you would like to make changes to your financial disclosure, please include your updated statement in your cover letter. Guidelines for resubmitting your figure files are available below the reviewer comments at the end of this letter.

If applicable, we recommend that you deposit your laboratory protocols in protocols.io to enhance the reproducibility of your results. Protocols.io assigns your protocol its own identifier (DOI) so that it can be cited independently in the future. For instructions see: https://journals.plos.org/plosone/s/submission-guidelines#loc-laboratory-protocols. Additionally, PLOS ONE offers an option for publishing peer-reviewed Lab Protocol articles, which describe protocols hosted on protocols.io. Read more information on sharing protocols at https://plos.org/protocols?utm_medium=editorial-email&utm_source=authorletters&utm_campaign=protocols.

As the corresponding author, your ORCID iD is verified in the submission system and will appear in the published article. PLOS supports the use of ORCID, and we encourage all coauthors to register for an ORCID iD and use it as well. Please encourage your coauthors to verify their ORCID iD within the submission system before final acceptance, as unverified ORCID iDs will not appear in the published article. *Only*  the individual author can complete the verification step; PLOS staff *cannot*  verify ORCID iDs on behalf of authors.

We look forward to receiving your revised manuscript.

Kind regards,

Peng Zhang, Ph.D.

Academic Editor

PLOS One

Journal Requirements:

1. If the reviewer comments include a recommendation to cite specific previously published works, please review and evaluate these publications to determine whether they are relevant and should be cited. There is no requirement to cite these works unless the editor has indicated otherwise.

2. Please review your reference list to ensure that it is complete and correct. If you have cited papers that have been retracted, please include the rationale for doing so in the manuscript text, or remove these references and replace them with relevant current references. Any changes to the reference list should be mentioned in the rebuttal letter that accompanies your revised manuscript. If you need to cite a retracted article, indicate the article’s retracted status in the References list and also include a citation and full reference for the retraction notice.

[Note: HTML markup is below. Please do not edit.]

Reviewers' comments:

Reviewer's Responses to Questions

**Comments to the Author**

1. If the authors have adequately addressed your comments raised in a previous round of review and you feel that this manuscript is now acceptable for publication, you may indicate that here to bypass the “Comments to the Author” section, enter your conflict of interest statement in the “Confidential to Editor” section, and submit your "Accept" recommendation.

Reviewer #1: All comments have been addressed

Reviewer #2: All comments have been addressed

**********

2. Is the manuscript technically sound, and do the data support the conclusions?

The manuscript must describe a technically sound piece of scientific research with data that supports the conclusions. Experiments must have been conducted rigorously, with appropriate controls, replication, and sample sizes. The conclusions must be drawn appropriately based on the data presented.

Reviewer #1: Yes

Reviewer #2: Yes

**********

3. Has the statistical analysis been performed appropriately and rigorously?

Reviewer #1: Yes

Reviewer #2: Yes

**********

4. Have the authors made all data underlying the findings in their manuscript fully available?

The PLOS Data policy requires authors to make all data underlying the findings described in their manuscript fully available without restriction, with rare exception (please refer to the Data Availability Statement in the manuscript PDF file). The data should be provided as part of the manuscript or its supporting information, or deposited to a public repository. For example, in addition to summary statistics, the data points behind means, medians and variance measures should be available. If there are restrictions on publicly sharing data—e.g. participant privacy or use of data from a third party—those must be specified.

Reviewer #1: Yes

Reviewer #2: Yes

**********

5. Is the manuscript presented in an intelligible fashion and written in standard English?

PLOS ONE does not copyedit accepted manuscripts, so the language in submitted articles must be clear, correct, and unambiguous. Any typographical or grammatical errors should be corrected at revision, so please note any specific errors here.

Reviewer #1: Yes

Reviewer #2: Yes

**********

6. Review Comments to the Author

Please use the space provided to explain your answers to the questions above. You may also include additional comments for the author, including concerns about dual publication, research ethics, or publication ethics. (Please upload your review as an attachment if it exceeds 20,000 characters)

Reviewer #1: Minor revision:

The letter p should be italicized throughout the statistical analysis section.

Please provide the full name of SOD at its first mention in the manuscript.

The manuscript should use a consistent and standard format for group names. For example, “Group A, Group B, Group C, Group D” is preferred over “A/B/C/D group” for clarity and consistency.

Line 314: The word “and” is used redundantly and should be removed.

The manuscript would benefit from careful language revision by a native English speaker to improve clarity and readability.

Reviewer #2: The authors have addressed all major concerns, clearly defined the groups, and significantly improved the clarity of the paper.

**********

7. PLOS authors have the option to publish the peer review history of their article (what does this mean?). If published, this will include your full peer review and any attached files.

If you choose “no”, your identity will remain anonymous but your review may still be made public.

**Do you want your identity to be public for this peer review?**  For information about this choice, including consent withdrawal, please see our Privacy Policy.

Reviewer #1: No

Reviewer #2: No

**********

[NOTE: If reviewer comments were submitted as an attachment file, they will be attached to this email and accessible via the submission site. Please log into your account, locate the manuscript record, and check for the action link "View Attachments". If this link does not appear, there are no attachment files.]

To ensure your figures meet our technical requirements, please review our figure guidelines: https://journals.plos.org/plosone/s/figures

You may also use PLOS’s free figure tool, NAAS, to help you prepare publication quality figures: https://journals.plos.org/plosone/s/figures#loc-tools-for-figure-preparation.

NAAS will assess whether your figures meet our technical requirements by comparing each figure against our figure specifications.

# Author comment: Author response to Decision Letter 3

**DOI:** 10.1371/journal.pone.0351145.r006

17 May 2026

Dear Dr. Zhang and Reviewers,

We sincerely thank the Academic Editor and the reviewers for their careful evaluation of our revised manuscript. We are grateful that both reviewers considered that the major concerns had been addressed. In response to the remaining minor comments from Reviewer #1, we have revised the manuscript and checked the full text, statistical notation, group-name formatting, figure legends, and language. A point-by-point response is provided below. All revisions are reflected in the revised manuscript and the marked-up version.

Response to Reviewer #1

We thank Reviewer #1 for the careful evaluation of our revised manuscript and for the constructive minor suggestions.

Comment 1: The letter p should be italicized throughout the statistical analysis section.

Response: Thank you for this suggestion. We have italicized the letter p throughout the Statistical analysis section. We also checked the Results section and figure legends to ensure consistent formatting of statistical expressions, including p-values.

Comment 2: Please provide the full name of SOD at its first mention in the manuscript.

Response: Thank you. We have revised the first mention of this abbreviation to “superoxide dismutase (SOD)” and used “SOD” thereafter. This revision is also reflected consistently in the relevant methods subsection and figure legend.

Comment 3: The manuscript should use a consistent and standard format for group names. For example, “Group A, Group B, Group C, Group D” is preferred over “A/B/C/D group” for clarity and consistency.

Response: We agree with the reviewer. We have carefully checked the manuscript and figure legends and standardized the group designations throughout the text. The groups are now described consistently as Group C, Group D, and Group D + GSK2795039, with definitions provided at first mention as follows: Group C, control group; Group D, decompression-stress group; and Group D + GSK2795039, NOX2 inhibitor-treated decompression-stress group.

Comment 4: Line 314: The word “and” is used redundantly and should be removed.

Response: Thank you for pointing this out. We have removed the redundant wording in the Conclusions section and revised the sentence to improve clarity and readability.

Comment 5: The manuscript would benefit from careful language revision by a native English speaker to improve clarity and readability.

Response: Thank you for this helpful suggestion. We have carefully revised the manuscript throughout for language, grammar, punctuation, terminology, clarity, and readability. We also checked the consistency of statistical expressions, group labels, and figure legends.

Response to Reviewer #2

Comment: The authors have addressed all major concerns, clearly defined the groups, and significantly improved the clarity of the paper.

Response: We sincerely thank Reviewer #2 for the positive assessment. No additional concerns were raised. We have retained the revisions that addressed the major concerns and conducted an additional final check to ensure consistency, clarity, and readability throughout the manuscript.

We hope that the revised manuscript and the responses above satisfactorily address the remaining comments. We again thank the Academic Editor and reviewers for their time and constructive feedback.

Sincerely,

Wang Song

Exercise Immunology Center, Wuhan Sports University

Wuhan, China

Attachment Submitted filename: Response_to_Reviewers_auresp_3.docx

# Peer review: Decision Letter 3

**DOI:** 10.1371/journal.pone.0351145.r007

**Authors:** Peng Zhang

24 May 2026

NOX2 inhibition attenuates oxidative stress and eNOS uncoupling in pulmonary arteries of rats following simulated air diving

PONE-D-25-62136R3

Dear Dr. Wang,

We’re pleased to inform you that your manuscript has been judged scientifically suitable for publication and will be formally accepted for publication once it meets all outstanding technical requirements.

Within one week, you’ll receive an e-mail detailing the required amendments. When these have been addressed, you’ll receive a formal acceptance letter and your manuscript will be scheduled for publication.

An invoice will be generated when your article is formally accepted. Please note, if your institution has a publishing partnership with PLOS and your article meets the relevant criteria, all or part of your publication costs will be covered. Please make sure your user information is up-to-date by logging into Editorial Manager at Editorial Manager® and clicking the ‘Update My Information' link at the top of the page. For questions related to billing, please contact billing support.

If your institution or institutions have a press office, please notify them about your upcoming paper to help maximize its impact. If they’ll be preparing press materials, please inform our press team as soon as possible -- no later than 48 hours after receiving the formal acceptance. Your manuscript will remain under strict press embargo until 2 pm Eastern Time on the date of publication. For more information, please contact onepress@plos.org.

Kind regards,

Peng Zhang, Ph.D.

Academic Editor

PLOS One

Additional Editor Comments (optional):

Reviewers' comments:

Reviewer's Responses to Questions

**Comments to the Author**

1. If the authors have adequately addressed your comments raised in a previous round of review and you feel that this manuscript is now acceptable for publication, you may indicate that here to bypass the “Comments to the Author” section, enter your conflict of interest statement in the “Confidential to Editor” section, and submit your "Accept" recommendation.

Reviewer #1: All comments have been addressed

**********

2. Is the manuscript technically sound, and do the data support the conclusions?

The manuscript must describe a technically sound piece of scientific research with data that supports the conclusions. Experiments must have been conducted rigorously, with appropriate controls, replication, and sample sizes. The conclusions must be drawn appropriately based on the data presented.

Reviewer #1: Yes

**********

3. Has the statistical analysis been performed appropriately and rigorously?

Reviewer #1: Yes

**********

4. Have the authors made all data underlying the findings in their manuscript fully available?

The PLOS Data policy requires authors to make all data underlying the findings described in their manuscript fully available without restriction, with rare exception (please refer to the Data Availability Statement in the manuscript PDF file). The data should be provided as part of the manuscript or its supporting information, or deposited to a public repository. For example, in addition to summary statistics, the data points behind means, medians and variance measures should be available. If there are restrictions on publicly sharing data—e.g. participant privacy or use of data from a third party—those must be specified.

Reviewer #1: Yes

**********

5. Is the manuscript presented in an intelligible fashion and written in standard English?

PLOS ONE does not copyedit accepted manuscripts, so the language in submitted articles must be clear, correct, and unambiguous. Any typographical or grammatical errors should be corrected at revision, so please note any specific errors here.

Reviewer #1: Yes

**********

6. Review Comments to the Author

Please use the space provided to explain your answers to the questions above. You may also include additional comments for the author, including concerns about dual publication, research ethics, or publication ethics. (Please upload your review as an attachment if it exceeds 20,000 characters)

Reviewer #1: (No Response)

**********

7. PLOS authors have the option to publish the peer review history of their article (what does this mean?). If published, this will include your full peer review and any attached files.

If you choose “no”, your identity will remain anonymous but your review may still be made public.

**Do you want your identity to be public for this peer review?**  For information about this choice, including consent withdrawal, please see our Privacy Policy.

Reviewer #1: No

**********

# Editor report: Acceptance letter

**DOI:** 10.1371/journal.pone.0351145.r008

**Authors:** Peng Zhang

PONE-D-25-62136R3

PLOS One

Dear Dr. Wang,

I'm pleased to inform you that your manuscript has been deemed suitable for publication in PLOS One. Congratulations! Your manuscript is now being handed over to our production team.

At this stage, our production department will prepare your paper for publication. This includes ensuring the following:

* All references, tables, and figures are properly cited

* All relevant supporting information is included in the manuscript submission,

* There are no issues that prevent the paper from being properly typeset

You will receive further instructions from the production team, including instructions on how to review your proof when it is ready. Please keep in mind that we are working through a large volume of accepted articles, so please give us a few days to review your paper and let you know the next and final steps.

Lastly, if your institution or institutions have a press office, please let them know about your upcoming paper now to help maximize its impact. If they'll be preparing press materials, please inform our press team within the next 48 hours. Your manuscript will remain under strict press embargo until 2 pm Eastern Time on the date of publication. For more information, please contact onepress@plos.org.

You will receive an invoice from PLOS for your publication fee after your manuscript has reached the completed accept phase. If you receive an email requesting payment before acceptance or for any other service, this may be a phishing scheme. Learn how to identify phishing emails and protect your accounts at https://explore.plos.org/phishing.

If we can help with anything else, please email us at customercare@plos.org.

Thank you for submitting your work to PLOS ONE and supporting open access.

Kind regards,

PLOS ONE Editorial Office Staff

on behalf of

Prof. Peng Zhang

Academic Editor

PLOS One
